# Three New Species of Phytotelm-Breeding *Melanophryniscus* from the Atlantic Rainforest of Southern Brazil (Anura: Bufonidae)

**DOI:** 10.1371/journal.pone.0142791

**Published:** 2015-12-02

**Authors:** Marcos R. Bornschein, Carina R. Firkowski, Diego Baldo, Luiz F. Ribeiro, Ricardo Belmonte-Lopes, Leandro Corrêa, Sérgio A. A. Morato, Marcio R. Pie

**Affiliations:** 1 Programa de Pós-Graduação em Ecologia, Conservação e Manejo da Vida Silvestre, Instituto de Ciências Biológicas, Universidade Federal de Minas Gerais, Belo Horizonte, Minas Gerais, Brazil; 2 Departamento de Zoologia, Universidade Federal do Paraná, Curitiba, Paraná, Brazil; 3 Mater Natura—Instituto de Estudos Ambientais, Curitiba, Paraná, Brazil; 4 Laboratorio de Genética Evolutiva, Instituto de Biología Subtropical, Facultad de Ciencias Exactas Químicas y Naturales, Universidad Nacional de Misiones, Misiones, Argentina; 5 Escola de Saúde, Pontifícia Universidade Católica do Paraná, Curitiba, Paraná, Brazil; 6 Programa de Pós-Graduação em Zoologia, Departamento de Zoologia, Universidade Federal do Paraná, Curitiba, Paraná, Brazil; 7 STCP Engenharia de Projetos Ltda, Curitiba, Paraná, Brazil; Trier University, GERMANY

## Abstract

Three new species of *Melanophryniscus* are described from the Serra do Mar mountain range of the state of Santa Catarina, southern Brazil. All species are found at intermediate to high altitudes and share phytotelm-breeding as their reproductive strategy. The new species are distinguished from other phytotelm-breeding *Melanophryniscus* based on different combinations of the following traits: snout-vent length, presence of white and/or yellow spots on forearms, mouth, belly and cloaca, pattern and arrangement of warts, and presence and number of corneous spines. The discovery of these species in a rather restricted geographical area suggests that the diversity of phytotelm-breeding species of *Melanophryniscus* might be severely underestimated. The conservation status of these species is of particular concern, given that one of them is at risk of extinction not only due to its restricted habitat, but also because of anthropogenic disturbances.

## Introduction

*Melanophryniscus* Gallardo, 1961 currently includes 26 toad species distributed from southeastern Brazil to Uruguay, central and northern Argentina, Paraguay, and southern Bolivia [1]. There is increasing phylogenetic evidence supporting the placement of *Melanophryniscus* as the sister group of the rest of Bufonidae (e.g. [2, 3]). Some species of *Melanophryniscus* have been shown to possess skin toxins, such as lipophilic alkaloids, which are obtained through their diet ([4]; see also [5, 6]. The current richness of the genus is likely underestimated, and the collection of new specimens and genetic analyses are expected to reveal new species [7, 8].

Species of *Melanophryniscus* have been traditionally divided into the *M*. *tumifrons*, *M*. *stelzneri*, and *M*. *moreirae* phenetic species groups [9], originally with eight, eight, and two species, respectively. The presence of a frontal macrogland (*sensu* [10]) has been proposed as a diagnostic character for the *M*. *tumifrons* group [9]. This character was later considered as a putative synapomorphy for the *M*. *tumifrons* group [11]. Additional support for this group was recently obtained based on chromosomal data [12]. The *M*. *moreirae* group was defined as species having “very developed warts with an apical corneous spine on dorsal surfaces and flanks, absence of conspicuous color pattern of contrasting spots or blotches on dorsum, and absence of a protuberance on the snout” [13]. Finally, the *M*. *stelzneri* group “involves species having corneous spines on shallow warts or directly on skin when these are absent, usually presence of conspicuous color pattern of contrasting spots or blotches on dorsum, and absence of a protuberance on the snout” [13]. Some cytogenetic characters support the *M*. *stelzneri* group [12].

Caramaschi and Cruz [9] emphasized that “several modifications are expected” in the *Melanophryniscus* species groups, given that they were erected without “modern taxonomical analysis” (see also [14]). For example, because of the absence of warts with keratinized spines, both *M*. *admirabilis* [14] and *M*. *alipioi* [15] do not match the characteristics of any of the Cruz and Caramaschi’s [13] species groups. Some of the new species of *Melanophryniscus* described after 2003 were not assigned into those species groups, namely *M*. *admirabilis* [14], *M*. *alipioi* [15], *M*. *vilavelhensis* [16], and *M*. *setiba* [17]. On the other hand, *M*. *krauczuki* [11], *M*. *paraguayensis* [18], and *M*. *estebani* [19] were placed in the *M*. *stelzneri* group, whereas *M*. *langonei* [20] was included in the *M*. *moreirae* group and *M*. *peritus* [21] was included in the *M*. *tumifrons* group. The placement of *M*. *krauczuki* as part of *M*. *stelzneri* group, however, was later questioned based on cytogenetic data [12]. Also, following other authors (e.g. [22]; see also [23]), Kwet *et al*. [7] suggested the removal of *M*. *rubriventris* from the *M*. *stelzneri* group [9] to establish its own group due to its larger size, strongly glandular skin (large granules), and blackish dorsal coloration. Finally, preliminary results of an ongoing molecular phylogenetic study of all species in the genus [24] indicate that *M*. *moreirae* forms a clade only with *M*. *alipioi* and *M*. *vilavelhensis*.

Recently, two new species of *Melanophryniscus* were described from the state of Paraná, in southern Brazil [15, 16]. Both are phytotelm-breeding species, which was a new reproductive mode for the genus [15]. Field expeditions from southern São Paulo state to eastern Santa Catarina state have revealed several new populations of phytotelm-breeding *Melanophryniscus* that were possibly overlooked previously due to their occurrence in high-altitude forests and grasslands (MRB *et al*. pers. obs.; this study). In the present study, we describe three new phytotelm-breeding species of *Melanophryniscus* from southern Brazil and discuss the implications of these discoveries for the conservation of the genus in the Atlantic Rainforest.

## Materials and Methods

This study was conducted with Brazilian research and collection permits, which include national (SISBio 22470–1, 22470–2, and 20416–2) and regional permits, from Instituto Ambiental do Paraná (355/11 and 07.15) and Fundação Municipal do Meio Ambiente of Joinville (permit 001/11), and follow the national guidelines regarding the collection and preservation of specimens for biological research and were approved as part of obtaining the field permits. Specimens were collected by hand, killed in an anesthetic solution, fixed in 10% formalin, and preserved in 70% ethyl alcohol. Measurements, taken with a caliper at a precision of 0.05 mm, are abbreviated as follows: SVL (snout-vent length), HL (head length from tip of snout to angle of jaw), HW (head width at widest point), ED (eye diameter), IOD (interorbital distance between anterior corners of the eyes), IND (internostril distance between inner margins of nostrils), END (eye-nostril distance—from anterior corner of the eye to posterior margin of nostril), THL (thigh length), TBL (tibia length), and FL (foot length).

Specimens were deposited at DZUP (Coleção de Herpetologia, Departamento de Zoologia, Universidade Federal do Paraná, Curitiba, state of Paraná, Brazil) and MHNCI (Museu de História Natural Capão da Imbuia, Prefeitura Municipal de Curitiba, Curitiba, state of Paraná, Brazil). Appendix I provides a complete list of examined specimens, which are deposited in the following institutions: BMNH = British Museum Natural History; CENAI (MACN) = Centro Nacional de Investigaciones Biológicas (now within MACN collection); CFBH = Célio F. B. Haddad collection, Departamento de Zoologia, Universidade Estadual Paulista, Rio Claro, state of São Paulo, Brazil; CHUNAM = Universidad Nacional de Misiones, Posadas, Argentina; FML = Fundación Miguel Lillo, Instituto de Herpetología, Tucumán, Argentina; HB = ex Colección herpetológica del Centro de Ecología aplicada del Litoral (now in MACN); MACN = Museo Argentino de Ciencias Naturales Bernardino Rivadavia, Buenos Aires, Argentina; MCP = Museu de Ciências e Tecnologia da Pontifícia Universidade Católica do Rio Grande do Sul, Porto Alegre, state of Rio Grande do Sul, Brazil; MFA-ZV.H = Museo Florentino Ameghino, Santa Fe, Argentina; MHNP = Museo de Historia Natural Del Paraguay, Asunción, Paraguay; MLP = Museo de La Plata, Argentina; MLP DB = Museo de la Plata, personal collection of D. Baldo, La Plata, Argentina; MNHN = Museo Nacional de Historia Natural, Montevideo, Uruguay; MNRJ = Museu Nacional, Rio de Janeiro, state of Rio de Janeiro, Brazil; MRCN = Museu de Ciências Naturais da Fundação Zoobotânica do Rio Grande do Sul, Porto Alegre, state of Rio Grande do Sul, Brazil; MZUSP = Museu de Zoologia da Universidade de São Paulo, São Paulo, state of São Paulo, Brazil; ZUFSM = Universidade Federal de Santa Maria, Santa Maria, state of Rio Grande do Sul, Brazil.

Adult individuals were considered males if at least one of the following criteria were met: (1) when they were found vocalizing; (2) when the specimen exhibited developed nuptial pads and/or vocal sac; or (3) when the male possessed mature testes (inspected by dissection). Likewise, adult individuals were considered females if (1) they were collected in amplexus with a male; (2) if the female released eggs; or (2) if the specimen possessed oocytes in the coelomic cavity (under dissection). Individuals were considered juveniles when they did not show any of the abovementioned secondary sexual characters or presented undifferentiated gonads (under dissection). Habitat descriptions followed the Brazilian vegetation classification system [25]. As indicated in Fabrezi and Alberch [26], fingers were numbered II-V. Descriptions of color in life followed the color numbers provided in Krüppers [27]. Geographic coordinates are based on the WGS84 datum. For the conservation status of the species and also to determine its “area of occupancy”, we followed the IUCN criteria [28].

### Nomenclatural acts

The electronic edition of this article conforms to the requirements of the amended International Code of Zoological Nomenclature, and hence the new names contained herein are available under that Code from the electronic edition of this article. This published work and the nomenclatural acts it contains have been registered in ZooBank, the online registration system for the ICZN. The ZooBank LSIDs (Life Science Identifiers) can be resolved and the associated information viewed through any standard web browser by appending the LSID to the prefix “http://zoobank.org/”. The LSID for this publication is: urn:lsid:zoobank.org:pub: 5876B4D8-4267-49C3-BF4C-7BA55A634816. The electronic edition of this work was published in a journal with an ISSN, and has been archived and is available from the following digital repositories: PubMed Central and LOCKSS.

## Results

### *Melanophryniscus biancae* sp. nov.

(Figs 1–3)

**Fig 1:**
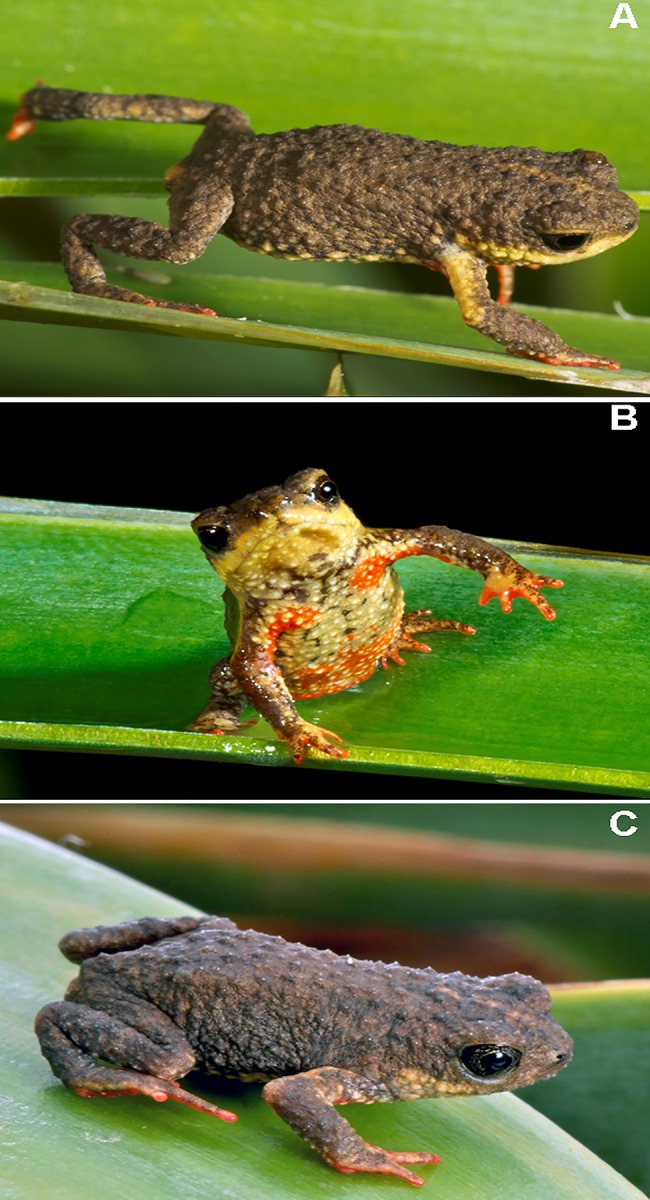
*Melanophryniscus biancae* sp. nov. Two adult males from the type-locality (Serra do Quiriri, municipality of Garuva): A, B = MHNCI 9809 (photographs by H. Garcia); C = MHNCI 9810.

**Fig 2:**
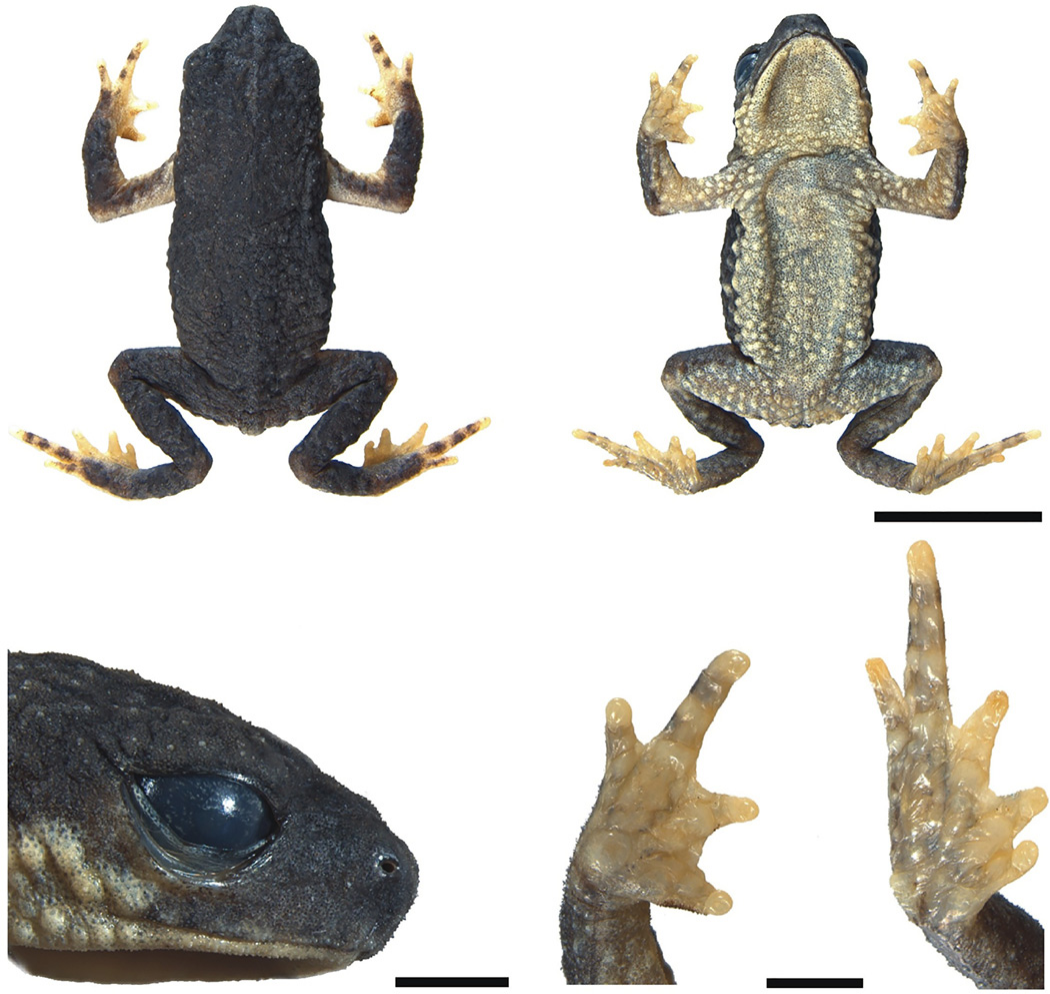
Holotype of *Melanophryniscus biancae* sp. nov. (DZUP 238), adult male. The lower surface of the right hand and right foot are shown on the bottom right. The scale bars on the top two photographs correspond to 0.5 cm, whereas on the bottom they correspond to 1 mm.

**Fig 3:**
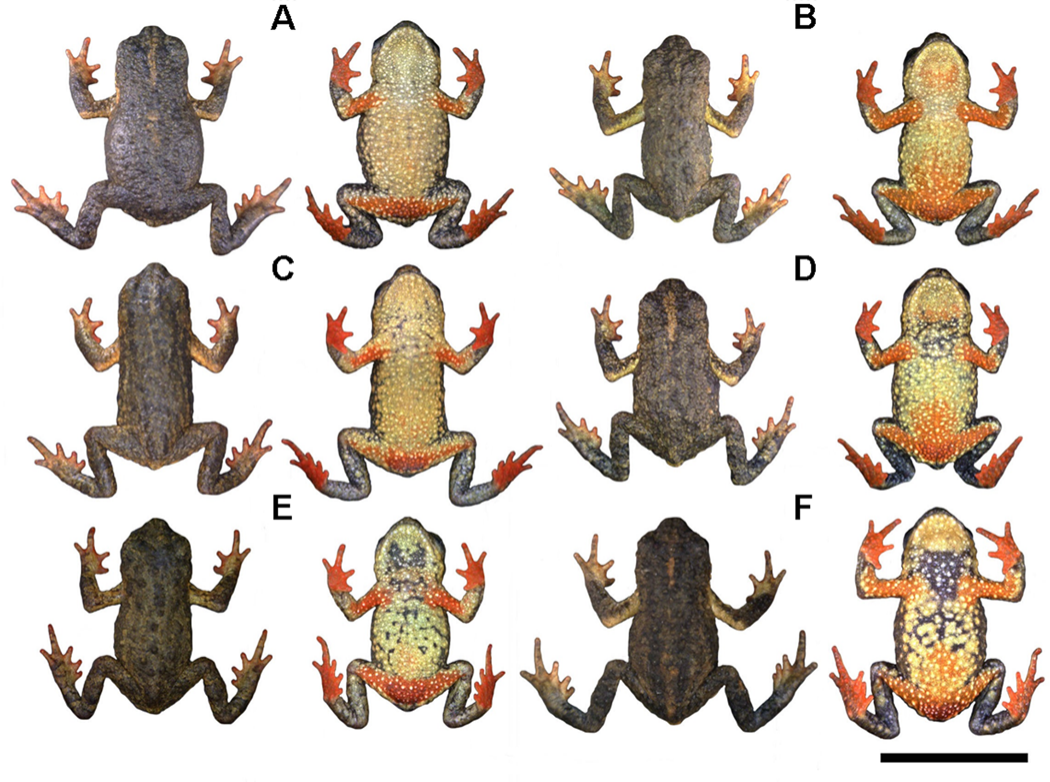
Representative variation in coloration in the type-series of *Melanophryniscus biancae* sp. nov., all adult males, alive, in dorsal and ventral view. A = DZUP 237; B = DZUP 238 (holotype); C = DZUP 240; D = DZUP 242; E = DZUP 239; F = DZUP 241. The bar corresponds to 1 cm.

M.R. Bornschein, D. Baldo, M.R. Pie, C.R. Firkowski, L.F. Ribeiro & L. Corrêa

Urn:lsid:zoobank.org:act: 44B4D7A6-A9BF-4EE0-85AC-D865CBB7336C

*M*. sp. 3 aff. *vilavelhensis* [24]

#### Holotype

DZUP 238 (Figs 2 and 3B), male, collected at Serra do Quiriri (26°01’42”S, 48°57’54”W; 1,310 m a.s.l.), municipality of Garuva, state of Santa Catarina, southern Brazil, on 15 February 2011 by MRB and LC.

#### Paratopotypes

DZUP 237, 239–42, 323–4 (Fig 3), seven males, all with the same data as the holotype, and DZUP 326, an unsexed juvenile, collected as an egg on the same day as the holotype, reared in the laboratory, killed and preserved on 21 July 2011. DZUP 231 and 325, male and one unsexed juvenile collected by MRB, MRP, and Dulce Carvalho on 3 February 2011 as eggs, which were reared in the laboratory, killed, and preserved on 21 July 2011.

#### Referred specimens

MHNCI 9809–10, two males, collected at the type locality by MRP and RB-L on 20 February 2015.

#### Diagnosis

The minute size of *M*. *biancae* sp. nov. (SVL = 12.9–13.8 mm) is closest to that of *M*. *vilavelhensis* (SVL = 12.8–17.2 mm, [16]) and *M*. *setiba* (SVL = 13.8–16.1 mm, [17]) and differs greatly from the remaining species: *M*. *admirabilis* (29.5–40.3 mm, [14]); *M*. *alipioi* (SVL = 19.4–27.8 mm); *M*. *atroluteus* (SVL = 18.3–25.7 mm); *M*. *cupreuscapularis* (SVL = 19.5–26.6 mm); *M*. *dorsalis* (SVL = 20.0–26.9 mm); *M*. *estebani* (SVL = 25.0–34.0 mm; [19]); *M*. *fulvoguttatus* (SVL = 19.7–27.2 mm); *M*. *krauczuki* (SVL = 18.0–24.4 mm); *M*. *langonei* (SVL = 20.5–21.5 mm; [20]); *M*. *montevidensis* (SVL = 18.6–28.0 mm); *M*. *moreirae* (SVL = 20.6–29.1 mm); *M*. *paraguayensis* (SVL = 20.5–25.6 mm, [18]); *M*. *rubriventris* (SVL = 32.0–42.67 mm); *M*. *sanmartini* (SVL = 19.0–24.9 mm); as well as from all species of the *M*. *tumifrons* group (always larger than 20 mm).

**Fig 4:**
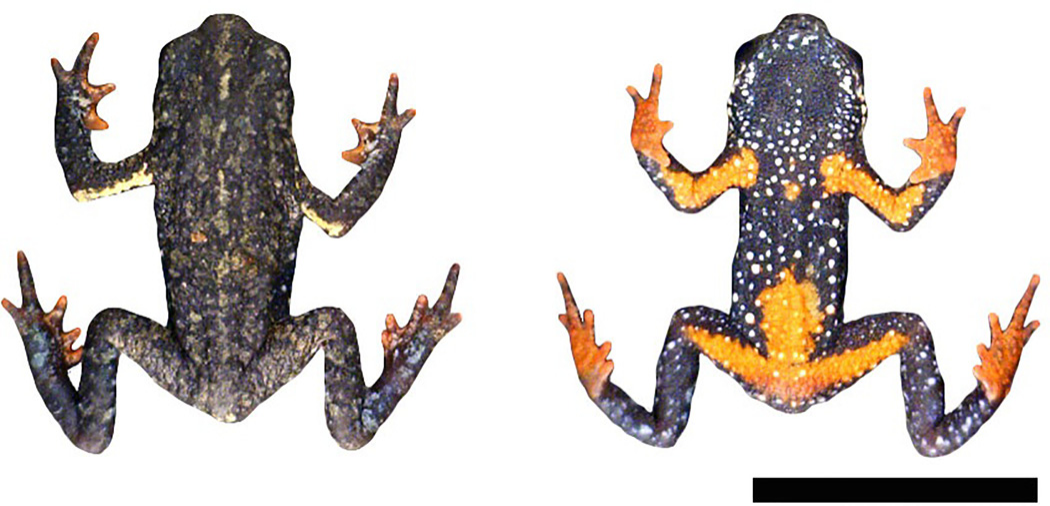
Adult male of *Melanophryniscus vilavelhensis* (DZUP 208) alive, in dorsal and ventral view, from the type locality of the species (Parque Estadual de Vila Velha, 25°14’50”S, 50°00’17”W, municipality of Ponta Grossa, Paraná, southern Brazil). The bar corresponds to 1 cm.

As to morphology, *M*. *biancae* sp. nov. is most similar to *M*. *vilavelhensis*, but the ventral surface of the former is almost completely yellow (Fig 3), whereas in the latter it is black with a small red area scattered with white dots (Fig 4). The predominantly brown dorsal surface of *M*. *biancae* sp. nov. (Fig 3) also differs from *M*. *vilavelhensis*, whose dorsal surface is predominantly black (Fig 4). *Melanophryniscus biancae* sp. nov. is easily distinguishable from *M*. *setiba* as follows (states of *M*. *setiba* between parentheses): dorsal skull elements not co-ossified (dorsal skull elements strongly co-ossified); fingers and toes unreduced (reduced phalangeal formula of both hands and feet); males without humeral spine externally visible (with an evident bifurcated humeral spine); males have a nuptial pad with several small brown-colored keratinized spines on fingers II and III (males with nuptial pad, with few enlarged, brown-colored keratinized spines at medial margin of finger II); head and dorsum skin with small glandular warts, tipped with apical spines (head and dorsum slightly granular without keratinous spines); ventral skin finely covered with glandular warts, tipped with spines, and entirely covered with tiny spicules (throat and chest smooth); dorsal coloration pattern marbled gray, brown, and gold with a lighter interocular spot and a white stripe covering posterior half of upper arm (dorsum reddish brown to dark brown with two dorsal marks, with the first mark on the anterior region shaped like an “X”, whereas the second mark is on the posterior region and is shaped like a “Ʌ”); ventral surface entirely orange with scattered white dots (light orange with large dark brown blotch at midbody).

**Fig 5:**
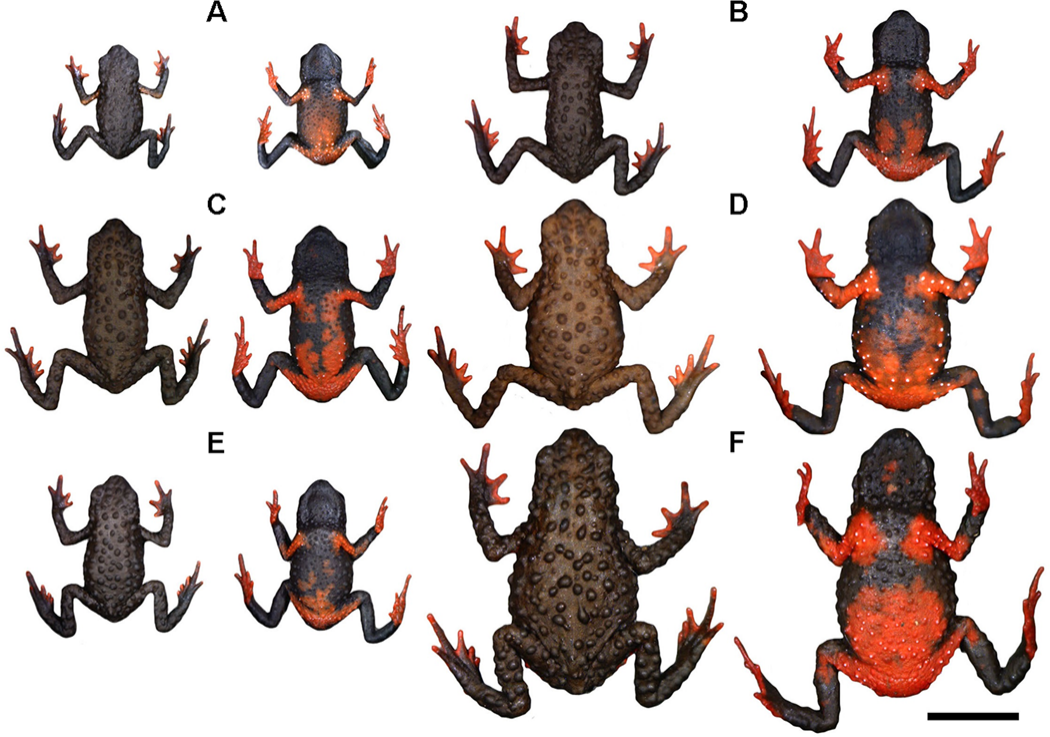
Representative variation in coloration in individuals of *Melanophryniscus alipioi*, alive, in dorsal and ventral view, from the type locality of the species (Capivari Grande, 25°07’49”S, 48°49’15”W, Serra do Capivari, municipality of Campina Grande do Sul, Paraná, southern Brazil). A = DZUP 284 (juvenile male); B = DZUP 293 (adult male); C = DZUP 290 (adult male); D = DZUP 285 (adult female); E = DZUP 292 (adult male); F = DZUP 291 (adult female). The bar corresponds to 1 cm.

The dorsal skin texture with small glandular warts tipped with apical spines differentiates *M*. *biancae* sp. nov. from *M*. *admirabilis* (skin with large, yellowish warts glands without apical spines), *M*. *alipioi* (dorsally rugose, scattered with medium to large, blunt, rounded glandular warts, without keratinized spines), *M*. *langonei* (dorsum rugose, with small to medium glandular warts, tipped with several apical spines and with several longitudinal glandular ridges), *M*. *moreirae* (smooth skin, covered with large glandular warts, devoid of apical spines in males and with only a few keratinized spines in females), *M*. *sanmartini* (dorsum rugose, with small to medium rounded glandular warts scattered, more abundantly in the head, and tipped with several apical spines), and from several species of the *M*. *stelzneri* group (skin almost smooth, with few developed glandular warts, tipped with few and small apical spines in *M*. *estebani*, *M*. *fulvoguttatus*, *M*. *klappenbachi*, and *M*. *stelzneri*; or skin smooth with large glandular warts devoid of apical spines in *M*. *rubriventris*). The dorsal coloration pattern with shades of brown color, patchily distributed or sometimes divided in side-by-side lines, separates *M*. *biancae* sp. nov. from *M*. *admirabilis* (almost homogeneously green to yellowish), *M*. *alipioi* (dark brown to black) (Fig 5), *M*. *krauczuki* (homogeneously dark brown), *M*. *sanmartini* (dark brown with light brown spots), *M*. *langonei* (light brown with the glandular ridges darker), *M*. *moreirae* (dark grey to black or brownish, with scattered lighter areas, particularly on the sides of the body), and from all species of the *M*. *stelzneri* group (black, dark brown or dark green, with copper, yellow, orange, or red spots in *M*. *atroluteus*, *M*. *cupreuscapularis*, *M*. *dorsalis*, *M*. *estebani*, *M*. *fulvoguttatus*, *M*. *klappenbachi*, *M*. *montevidensis*, *M*. *paraguayensis*, *M*. *rubriventris*, and *M*. *stelzneri*).

The ventral pattern yellow with scattered white dots also differentiates *M*. *biancae* sp. nov. from *M*. *alipioi* (Fig 5), *M*. *moreirae*, all species of *M*. *stelzneri* group (except *M*. *rubriventris*), and *M*. *admirabilis* (all dark brown or black, with a large red/orange femoral patch, and different patterns of red, orange, or yellow spots in abdominal, throat, gular, and pectoral areas). Finally, *M*. *biancae* sp. nov. is easily distinguishable from all species of the *M*. *tumifrons* group (*M*. *devincenzii*, *M*. *macrogranulosus*, *M*. *pachyrhynus*, *M*. *peritus*, *M*. *simplex*, *M*. *spectabilis*, and *M*. *tumifrons*) due to the lack of a frontal macrogland.

#### Description of holotype

Body stout; head almost as long as wide (HL/HW = 0.99), approximately one third of SVL (HL/SVL = 0.32); snout short, truncate in dorsal view, protruding in lateral profile; nostrils small, oval, not protruding, directed anterolaterally, and located near to anterior terminus of snout; frontal macrogland absent; loreal region nearly flat, vertical; eye large, pupil horizontally elliptical; eye diameter equal to half the interorbital distance (ED/IOD = 0.5); cephalic crests absent; parotoid gland absent; tympanic membrane and supratympanic fold absent; without evident co-ossification of the dorsal skull elements; premaxillary, maxillary, and vomerine teeth absent; tongue narrow and elongate, posterior margin entire, posteriorly free for about two thirds of length; widely separated, small and rounded choanae; vocal sac medial subgular, without evident external fold; without externally evident ventral humeral crest; arms and forearms robust; finger blunt, short and slightly webbed at the base, more extended between fingers II-III and III-IV; finger tips rounded; keratinized brown nuptial pads on finger II and proximal medial margin of finger III; relative lengths of fingers IV>V>III>II, inner tubercle metacarpal barely noticeable; outer metacarpal tubercle rounded; subarticular tubercles rounded; some rounded supernumerary palmar tubercles; legs short, slender; toes short, unreduced, slightly webbed at the base; toe tips rounded; relative lengths of toes IV>V = III>II>I; inner metatarsal tubercle undistinguished; outer metatarsal tubercle low, oval, and barely noticeable; subarticular tubercles oval, divided on finger IV; small and low rounded supernumerary plantar tubercles spaced from one another; dorsum skin scattered with blunt, rounded glandular warts; warts tipped with small, light keratinized spines; dorsal skin covered with small glandular warts, tipped with keratinized spines; ventral skin finely covered by glandular warts, tipped with light keratinized spines, and entirely covered with tiny spicules. Measurements of holotype (in mm) are: SVL = 13.1; HL = 4.2; HW = 4.3; ED = 1.5; IOD = 3.0; IND = 0.8; END = 1.2; THL = 4.7; TBL = 4.6; FL = 7.1.

#### Color in life

Dark brown (A60: M80/C90) back with a lateral stripe on each side, extending from the eye region to the thigh, brown (A69: M70/C80), and a small patch in the medial cephalic region light brown (A70: M70/C70) (Fig 3B). Arms, in dorsal view, brown (A69: M70/C80) with a faded yellow band (A80: M10/C00) extending from the armpit to the elbow. Legs, in dorsal view, with dark brown (A60: M80/C90) and brown (A69: M70/C80) bands. Hands and feet, in dorsal view, orange (A80: M60/C00) with brown stripes (A69: M70/C80) in the fingers; tip of the fingers orange-red (A70: M80/C00). Supracloacal region faded yellow (A80: M10/C00). Belly predominantly yellow (A40: M00/C00) with many dots on glandular warts of same yellow color, but that stand out seeming to be another tone by the absence of a more darkened background that exists in other parts of the belly, particularly between the arms (Fig 3B). Region of the vocal sac and abdomen yellow and red (A70: M70/C00); near the cloaca, dark red (A70: M90/C50). Arms, in ventral view, red (A70: M70/C00) with a few yellow dots (A40: M00/C00); close to hands, with a gray patch maculate with yellow and some white dots. Legs, in ventral view, red (A70: M70/C00) with yellow dots (A40: M00/C00) on the thighs and with a gray color washed with yellow, containing white dots, in the rest; part of the red region is bordered with yellow (A40: M00/C00). Hands and feet, in ventral view, orange-red (A70: M80/C00).

#### Variation in type series

The range of measurements of adult paratypes is shown in Table 1. Snout shape in dorsal view varies from truncated (DZUP 240–2, 245, 323–5) to slightly mucronate in some specimens (DZUP 237, 239, 326). Males have nuptial pads covering the entire dorsal surface of the finger II and the proximal half of the dorsal surface of the finger III (only on finger II in DZUP 326). The nuptial pad spines can be dark brown (DZUP 237–8, 342), light brown (DZUP 239–41, 323, 325), or unpigmented (DZUP 326). No female of *M*. *biancae* sp. nov. has been collected to date.

**Table 1:** Measurements of adults of *Melanophryniscus* spp. nov. Values are sample size (N), range, and mean ± standard deviation (in parentheses). Abbreviations: m = male; f = female.

Measure^1^	Sex	*M*. *biancae* sp. nov.	*M*. *milanoi* sp. nov.		*M*. *xanthostomus* sp. nov.
			Type locality	Additional localities	
		N = 9 m	N = 12 m	N = 31 m; N = 3 f	N = 9 m; N = 3 f
SVL	m	12.9–13.8 (13.39 ± 0.35	18.6–21.1 (19.30 ± 0.67)	17.6–21.1 (19.20 ± 0.73)	18.1–21.5 (20.36 ± 1.05)
	f	–	–	20.3–23.7 (22.18 ± 1.71)	20.5–21.5 (21.11 ± 0.56)
HL	m	4.1–4.8 (4.41 ± 0.31)	6.0–6.7 (6.31 ± 0.25)	5.0–6.7 (6.07 ± 0.36)	5.4–6.9 (6.10 ± 0.44)
	f	–	–	5.8–6.7 6.19 ± 0.50)	5.8–6.7 (6.33 ± 0.49)
HW	m	4.2–4.7 (4.46 ± 0.17)	6.4–6.9 (6.60 ± 0.20)	5.8–6.9 (6.46 ± 0.27)	5.8–7.1 (6.49 ± 0.45)
	f	–	–	6.9–7.2 (7.02 ± 0.15)	6.9–7.2 (7.03 ± 0.11)
ED	m	1.3–1.6 (1.45 ± 0.13)	1.7–2.1 (1.96 ± 0.11)	1.7–2.2 (1.96 ± 0.12)	1.7–2.2 (1.99 ± 0.16)
	f	–	–	1.7–2.1 (1.94 ± 0.23)	1.8–2.3 (2.05 ± 0.27)
IOD	m	2.4–3.0 (2.80 ± 0.20)	3.7–4.4 (4.01 ± 0.21)	3.3–4.4 (3.84 ± 0.26)	3.6–4.6 (4.10 ± 0.31)
	f	–	–	3.9–4.0 (3.97 ± 0.07)	4.0–4.7 (4.28 ± 0.34)
IND	m	0.7–1.0 (0.88 ± 0.09)	1.3–1.7 (1.49 ± 0.15)	1.2–1.7 (1.43 ± 0.12)	1.2–1.9 (1.55 ± 0.24)
	f	–	–	1.2–1.8 (1.55 ± 0.32)	1.4–1.7 (1.56 ± 0.16)
END	m	0.8–1.2 (0.98 ± 0.14)	1.5–2.1 (1.80 ± 0.16)	1.3–2.1 (1.64 ± 0.19)	1.5–2.1 (1.68 ± 0.20)
	f	–	–	1.3–1.8 (1.61 ± 0.25)	1.6–1.9 (1.78 ± 0.13)
THL	m	4.4–5.4 (5.01 ± 0.30)	6.9–7.8 (7.43 ± 0.25)	6.9–8.5 (7.67 ± 0.40)	7.0–8.7 (7.81 ± 0.69)
	f	–	–	8.9–9.1 (9.01 ± 0.09)	7.8–9.2 (8.54 ± 0.66)
TBL	m	4.4–5.0 (4.70 ± 0.21)	6.8–7.5 (7.11 ± 0.22)	6.2–7.5 (6.93 ± 0.26)	6.6–7.8 (7.25 ± 0.33)
	f	–	–	7.5–8.2 (7.78 ± 0.41)	7.4–8.2 (7.88 ± 0.44)
FL	m	6.8–7.8 (7.31 ± 0.34)	9.9–11.5 (10.85 ± 0.43)	9.3–11.7 10.66 ± 0.50)	9.8–12.5 (11.29 ± 0.88)
	f	–	–	11.5–12.5 (11.82 ± 0.61)	12.2–13.0 (12.54 ± 0.45)

^1^ SVL = snout-vent length; HL = head length (from tip of snout to angle of jaw); HW = head width (greatest width); ED = eye diameter; IOD = interorbital distance (between anterior corners of the eyes); IND = internostril distance (between inner margins of nostrils); END = eye-nostril distance (from anterior corner of the eye to posterior margin of nostril); THL = thigh length; TBL = tibia length; and FL = foot length.

Some variation is found in dorsal and ventral color patterns (Fig 3). The light brown patch on the medial cephalic region can be absent (DZUP 239); poorly defined (DZUP 238, 240, 324–5); extent from the anterior interocular region to behind of head (DZUP 237, 241–2); or extent from the posterior interocular region to behind of head (DZUP 323). The dorsal surface of the majority of the paratopotypes has both shades of brown colors not divided in side-by-side lines but patchily distributed. The yellow pattern of the upper surface of arms was little perceptible in DZUP 239. The supracloacal region was of a darker yellow (DZUP 237, 239) or lighter yellow (DZUP 242, 324). Ventrally, there is no trace of red in DZUP 237 and only a very limited tinge of red on DZUP 240. Some individuals had black ventral coloration in the gular, chest, and/or in the belly regions. A black ventral coloration was vestigial (DZUP 240) or conspicuous (DZUP 239, 241–2, 324–5). The red of the thighs was reduced on DZUP 240; the rest of the legs can be blackish (DZUP 242).

#### Etymology

The specific epithet honors Bianca Luiza Reinert, ornithologist and environmentalist who dedicated her life to protect wetlands of the state of Paraná, southern Brazil.

#### Natural history

**Fig 6:**
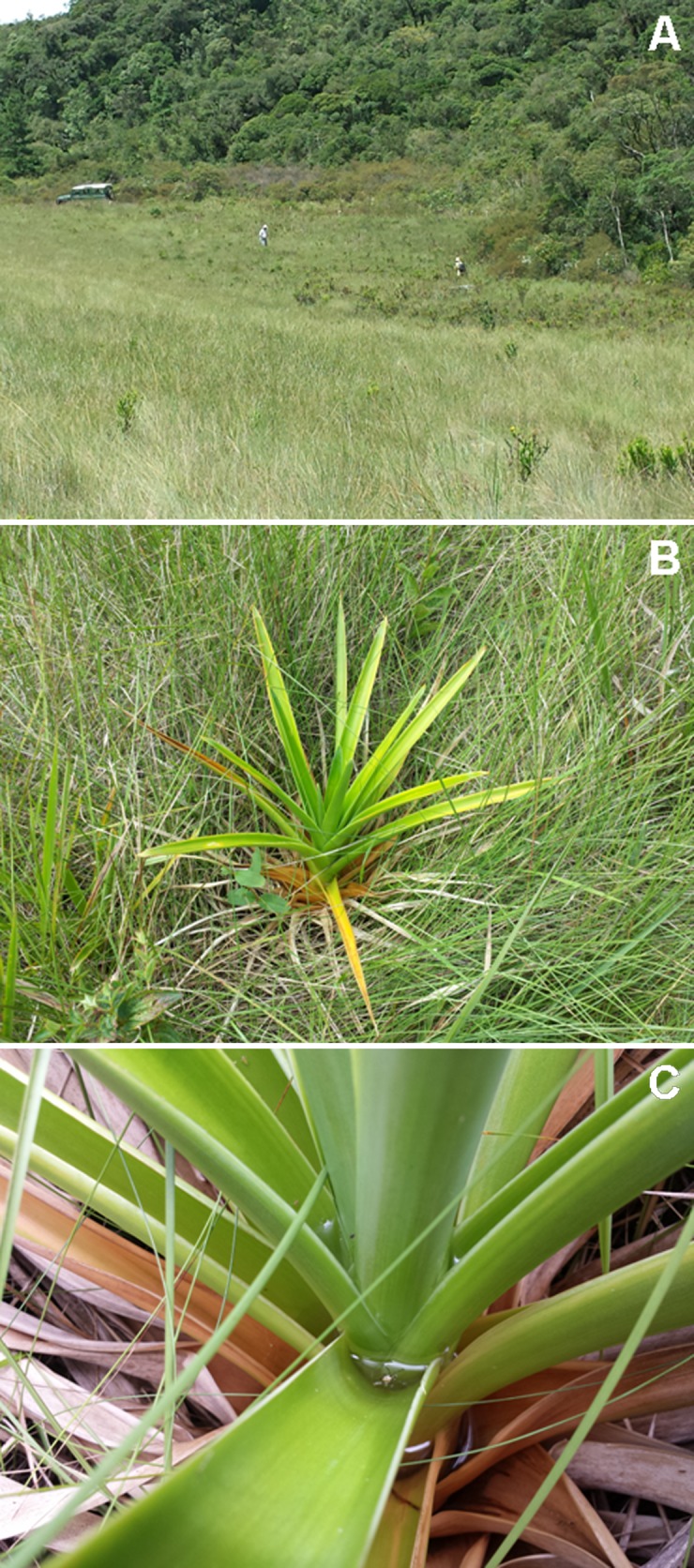
Habitat of *Melanophryniscus biancae* sp. nov. in the type-locality (Serra do Quiriri, municipality of Garuva). A = In the middle of the photograph, marshes where the species was found (two people are also present for scale)—in the foreground there are grasslands without occurrence of the plant where the species reproduces. B = *Eriocaulon ligulatum* (Eriocaulaceae) in marshes, the reproductive site of the new species. C = Base of leaves of *E*. *ligulatum* with water tanks where males sing and eggs are laid, except for the water tanks of the dead, yellow leaves.

*Melanophryniscus biancae* sp. nov. is a montane species (1,310–1,465 m a.s.l.) that inhabits marshes (“Formação Pioneira de Influência Fluvial”) that are present along small water courses or in small water drainages in natural grasslands (“Estepe Gramíneo Lenhosa”), at least during their reproductive season (Fig 6). We do not know if the species exclusively inhabits marshes, or if it occurs elsewhere but returns to these habitats during the reproductive season. Reproduction was observed to take place in *Eriocaulon ligulatum* (Eriocaulaceae; Fig 6B and 6C).

We detected males calling in *E*. *ligulatum* at night; they began to call 25 min after sunset and continued until at least 00 h 30 min on a cold night (13°C) with drizzle on 15 February 2011. On that occasion, we found eggs and a tadpole. On 3 February 2011 we found eggs, tadpoles and some specimens near the end of metamorphosis. On 9 October 2004 we found eggs. On 20 January and 20 February 2015, each following three days of rain, we did not find eggs and tadpoles in *E*. *ligulatum*. In particular, on the second date, in 2 h of searching after the sunset we found only two males in *E*. *ligulatum*.

We found eggs and tadpoles in the water at the base of green leaves of *E*. *ligulatum* that were either horizontal or slightly inclined upwards (Fig 6C). In one plant, we found some eggs in the small volume of water that accumulates near the base of vertical leaves, whereas in another plant we found some eggs in leaves 2 cm above the water. Eggs were isolated from each other and were found freely in the water or attached to the abaxial surface of leaves. We found one to four eggs in a water tank and found three to nine eggs per plant.

Collected eggs developed in an aquarium and individuals were transferred to a closed terrarium soon after completing metamorphosis, in which they developed until becoming adults (see above in Paratopotypes) without any additional food source. Individuals in the terrarium had nocturnal activity.

#### Geographical distribution

**Fig 7:**
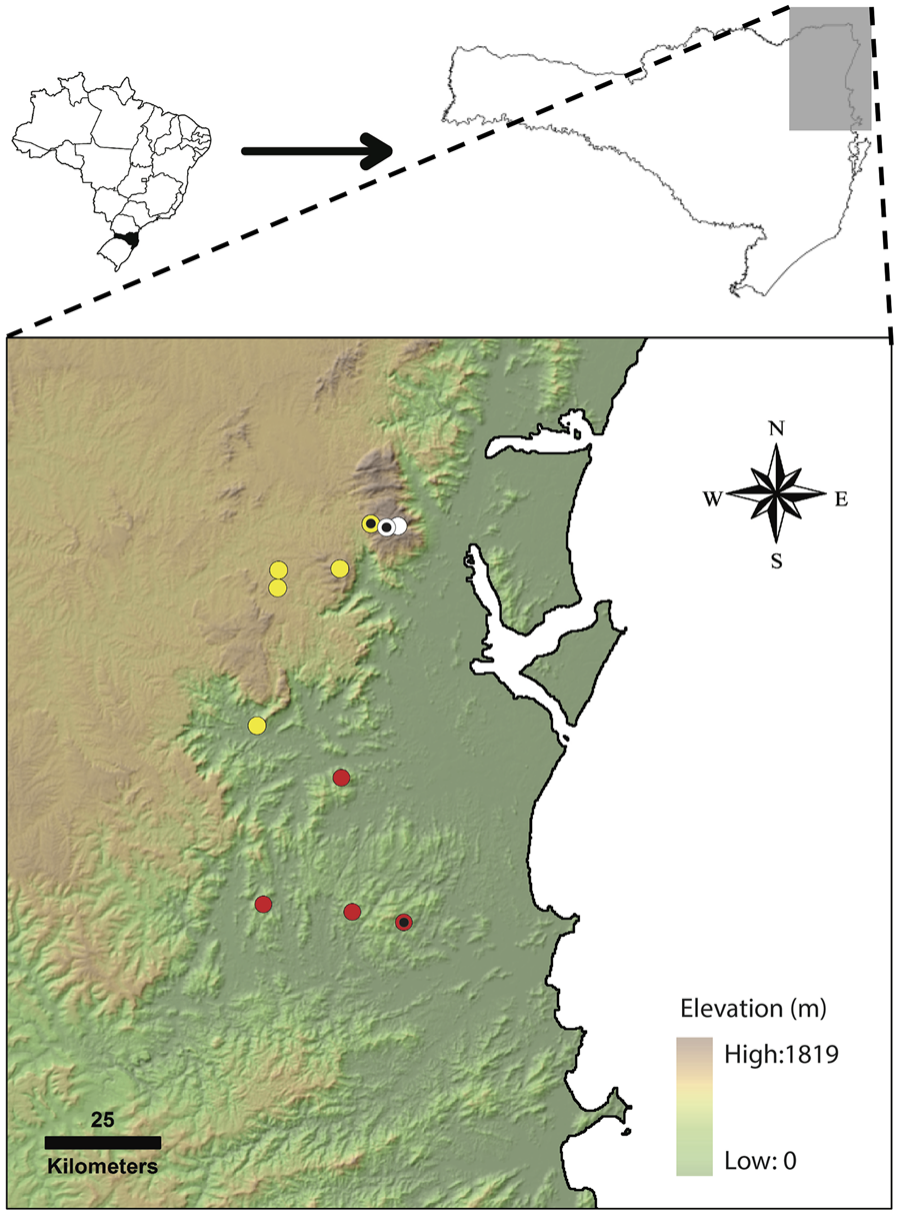
Geographical distribution of the new species of *Melanophryniscus* described in the present study. White, yellow, and red markers indicate occurrence records of *M*. *biancae* sp. nov., *M*. *xanthostomus* sp. nov., and *M*. *milanoi* sp. nov., respectively. Markers with black dots indicate type localities. Background based on SRTM30 elevation data.

The species is known from the type locality (a marsh with ~ 0.74 ha) and from a second locality in the same mountain range, owned by the Ciser company (26°01’33”S, 48°56’37”W; 1,465 m a.s.l.; Fig 7), Serra do Quiriri, municipality of Garuva, northeastern Santa Catarina, southern Brazil, where eggs were collected by J. A. Langone, R. O. de Sá, M. V. Segalla, and MRB on 9 October 2004. The eggs were kept in laboratory, hatched and 16 individuals metamorphosed between 16 and 20 November 2004 (DZUP uncatalogued). These two locations are distant from each other by only 2.1 km. We did not find the species in a marsh with *E*. *ligulatum* in the foothills of the Serra do Quiriri (municipality of Campo Alegre [26°00’20”S, 49°01’56”W; 842 m a.s.l.; 2.09 ha], state of Santa Catarina) on the same night of 15 February 2011 when the type series was collected.

### *Melanophryniscus milanoi* sp. nov.

(Figs 8–10)

**Fig 8:**
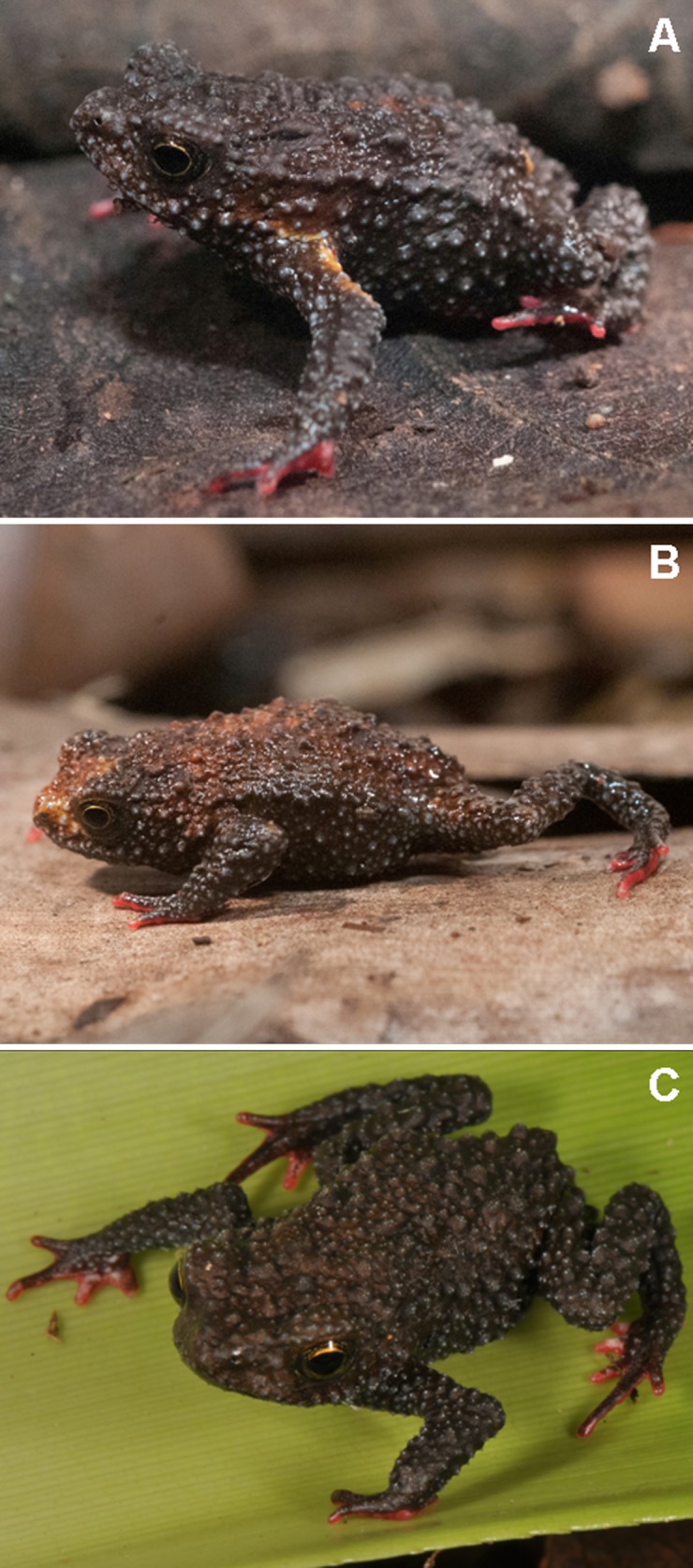
*Melanophryniscus milanoi* sp. nov. A = Adult male from Morro do Baú (DZUP 206); B = Adult male from Morro do Baú (DZUP 201); C = Adult male from Morro do Cachorro (DZUP 460).

**Fig 9:**
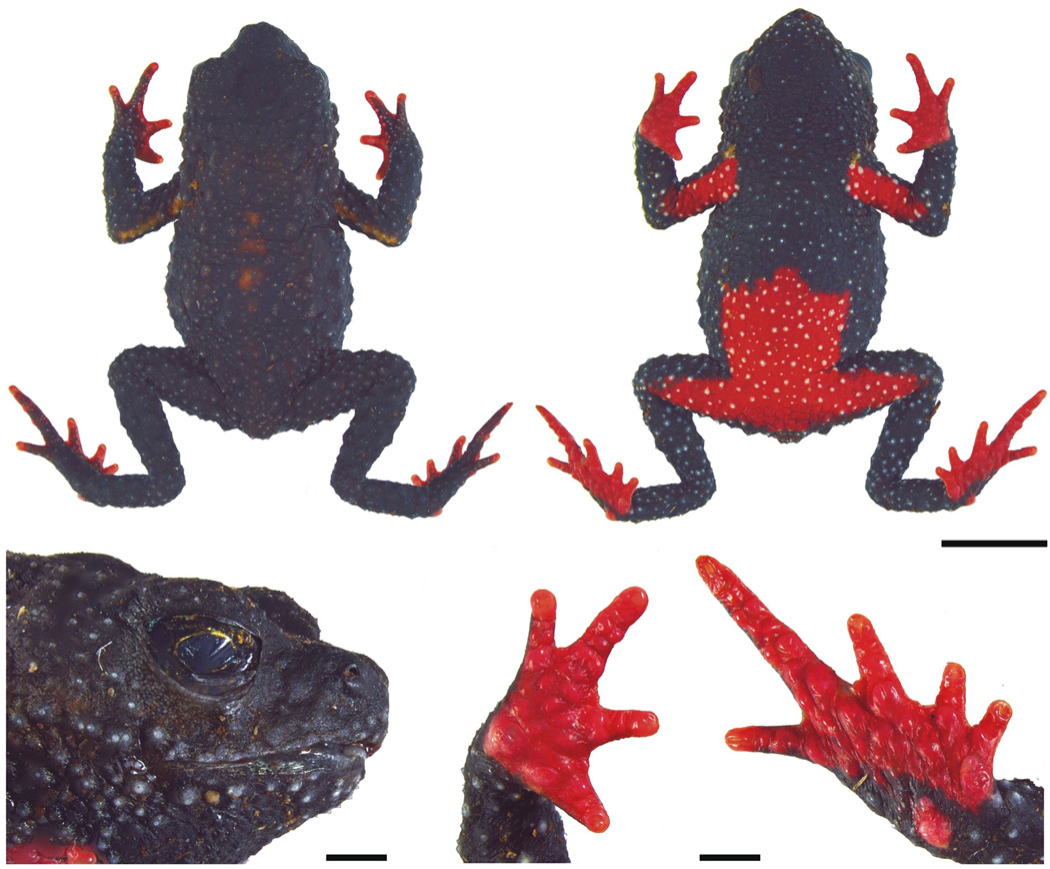
Holotype of *Melanophryniscus milanoi* sp. nov. (DZUP 205), adult male, a few minutes after being fixed. The lower surface of the right hand and right foot are shown on the bottom right. The scale bars on the top two photographs correspond to 0.5 cm, whereas on the bottom they correspond to 1 mm.

**Fig 10:**
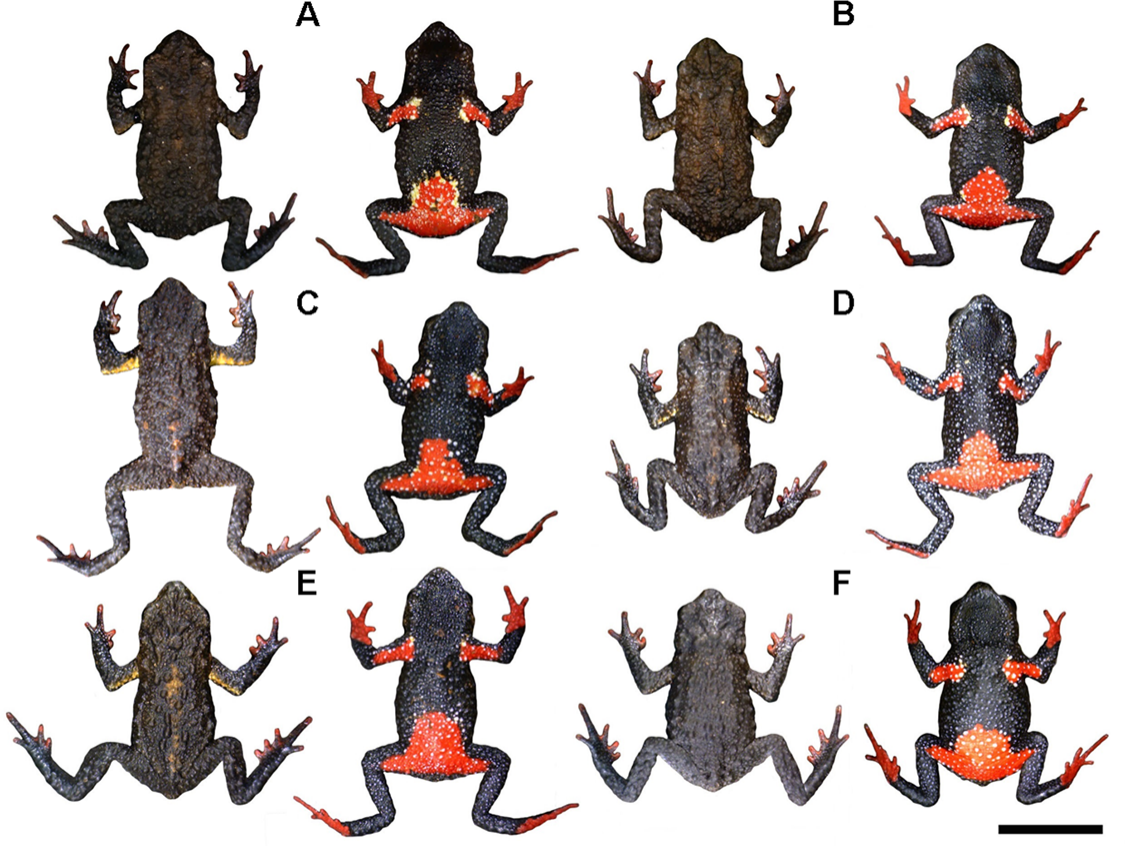
Representative variation in coloration in the type-series of *Melanophryniscus milanoi* sp. nov., all adult males, alive, in dorsal and ventral view. A = DZUP 199; B = DZUP 226; C = DZUP 227; D = DZUP 202; E = DZUP 203; F = DZUP 206. The bar corresponds to 1 cm.

D. Baldo, M.R. Bornschein, M.R. Pie, C.R. Firkowski, L.F. Ribeiro & R. Belmonte-Lopes

Urn:lsid:zoobank.org:act: BFD08F89-237F-40F1-962E-6353B79DFC6B

*M*. sp. 5 [24]

#### Holotype

DZUP 205 (Fig 9), male, collected at Morro do Baú (26°47’55”S, 48°55’55”W; 680 m a.s.l.), municipality of Ilhota, state of Santa Catarina, southern Brazil, on 26 October 2010 by MRB, RB-L, LC, MRP, and CRF.

#### Paratopotypes

DZUP 199, male, collected on 23 October 2010 by MRB and RB-L. DZUP 200–4, 206–7, 225–7 (Fig 10), 10 males, all with same data as holotype. All specimens collected between 655 and 710 m a.s.l.

#### Referred specimens

DZUP 418, 432–6, six males, collected at Morro Boa Vista (26°30’58”S, 49°03’14”W; 835–850 m a.s.l.), on the border between the municipalities of Jaraguá do Sul and Massaranduba, state of Santa Catarina, southern Brazil, on 25 October 2012 by MRP, LFR, and Felipe A. Cini da Silva. DZUP 417, female, and DZUP 427–31, five males, collected at Morro Azul (26°45’49”S, 49°12’23”W; 720–740 m a.s.l.), on the border between the municipalities of Pomerode and Rio dos Cedros, state of Santa Catarina, southern Brazil, on 26 October 2012 by MRP, LFR, and Felipe A. Cini da Silva. DZUP 416, 419–26, 437, 460, three females and eight males, collected at Morro do Cachorro (26°46’42”S, 49°01’57”W; 755–795 m a.s.l.), on the border between the municipalities of Blumenau, Gaspar, and Luiz Alves, state of Santa Catarina, southern Brazil, on 29 October 2012 by MRP, LFR, and Felipe A. Cini da Silva.

#### Diagnosis

Due to its intermediate size (SVL = 17.6–23.7 mm; Table 1), *M*. *milanoi* sp. nov. differs from the smaller (*M*. *vilavelhensis*, SVL = 12.8–17.2 mm, [16]; *M*. *setiba*, SVL = 13.8–16.1 mm, [17], and *M*. *biancae* sp. nov. SVL = 12.9–13.8 mm [see above]), and from larger species of the genus (*M*. *estebani*, SVL = 25.0–34.0 mm, [19]; *M*. *admirabilis*, 29.5–40.3 mm, [14]; and *M*. *rubriventris*, SVL = 32.0–42.7 mm).

The rugose dorsal skin, entirely covered with small to medium glandular warts uniformly scattered and tipped with several apical spines in *M*. *milanoi* sp. nov., allows it to be differentiated from *M*. *admirabilis* (skin with large, yellowish glandular warts without apical spines), *M*. *alipioi* (dorsally rugose, scattered with medium to large, blunt, rounded glandular warts, without keratinized spines; Fig 5), *M*. *langonei* (dorsum rugose with small to medium glandular warts, tipped with several apical spines and with several longitudinal glandular ridges), *M*. *moreirae* (smooth dorsal skin, covered with large glandular warts, devoid of apical spines in males and with only a few keratinized spines in females), *M*. *sanmartini* (dorsum rugose, with small to medium rounded glandular warts scattered, more abundantly in the head, and tipped with several apical spines), and from several species of the *M*. *stelzneri* group (skin almost smooth, with few developed glandular warts, tipped with few and small apical spines in *M*. *estebani*, *M*. *fulvoguttatus*, *M*. *klappenbachi*, and *M*. *stelzneri*; or skin smooth with large glandular warts devoid of apical spines in *M*. *rubriventris*).

The dorsal coloration pattern (with a mixture of two shades of brown patchily distributed, rarely with a yellow stripe on upper arms) also differentiates *M*. *milanoi* sp. nov. from *M*. *admirabilis* (almost homogeneously green to yellowish), *M*. *alipioi* (almost homogeneously brown to blackish, without a yellow stripe on upper arms; Fig 5), *M*. *biancae* sp. nov. (shades of brown sometimes divided in side-by-side lines and always with a yellow stripe on upper arms; Fig 3), *M*. *krauczuki* (homogeneously dark brown, without yellow stripe on upper arms), *M*. *langonei* (dorsal color light brown with the glandular ridges darker), *M*. *moreirae* (dorsum dark grey to black or brownish with scattered light areas with a white stripe on upper arms and a supracloacal white spot), and from all species of the *M*. *stelzneri* group (black, dark brown or dark green dorsal coloration, with copper, yellow, orange or red spots in *M*. *atroluteus*, *M*. *cupreuscapularis*, *M*. *dorsalis*, *M*. *estebani*, *M*. *fulvoguttatus*, *M*. *klappenbachi*, *M*. *montevidensis*, *M*. *paraguayensis*, *M*. *rubriventris*, and *M*. *stelzneri*).

The black ventral pattern, scattered with small white dots, a red patch covering the femoral region and the posterior half of belly, and throat, gular and pectoral regions spotless differentiate *M*. *milanoi* sp. nov. from *M*. *alipioi* (Fig 5) and *M*. *moreirae* (dark brown with bright red in the pectoral, abdominal, and femoral areas; red spots densely dotted with small white dots in throat, gular and pectoral areas in both species), from *M*. *biancae* sp. nov. (almost entirely yellow; Fig 3), *M*. *admirabilis* (black; gular region with large yellow glands; chest and belly with irregular pale green or yellowish; femoral region with a red patch), *M*. *langonei* (gular and pectoral zones uniformly black, belly black with large, reticulated orange-red spots, and a red patch covering the femoral region), *M*. *krauczuki* (dark brown; ventral of head with numerous small white dots along the mandibular arch; pectoral region with small white dots; belly with large, irregular, and bright orange spots; femoral region with a orange patch), *M*. *sanmartini* (brown to black; femoral region red/orange; pectoral and abdominal region with red/orange/yellow spots; gular region brown with a small white spot on chin), and from all species of *M*. *stelzneri* group (dark with yellow, orange or red irregular spots in throat, gular, pectoral, and abdominal regions; red/orange femoral region).

By its mucronate snout shape in dorsal view, *M*. *milanoi* sp. nov. also differs from *M*. *moreirae* (nearly rounded). *Melanophryniscus milanoi* sp. nov. is readily distinguishable from *M*. *setiba* as follows (states of *M*. *setiba* between parentheses): dorsal skull elements not co-ossified (dorsal skull elements strongly co-ossified); fingers and toes unreduced (reduced phalangeal formula of both hands and feet); males without humeral spine externally visible (with an evident bifurcated humeral spine); males have a nuptial pad with several small brown-colored keratinized spines on fingers II and III (males with nuptial pad with few enlarged, brown-colored keratinized spines at medial margin of finger II); dorsum skin with glandular warts tipped with apical spines (slightly granular without keratinous spines; throat and chest smooth); dorsal color pattern homogeneously brown (reddish brown to dark brown with two dorsal marks, with the first mark on the anterior region shaped like an “X”, whereas the second mark is on the posterior region and is shaped like a “Ʌ”). Finally, *M*. *milanoi* sp. nov. is easily distinguishable from all species of the *M*. *tumifrons* group (*M*. *devincenzii*, *M*. *macrogranulosus*, *M*. *pachyrhynus*, *M*. *peritus*, *M*. *simplex*, *M*. *spectabilis*, and *M*. *tumifrons*) due to the lack of a frontal macrogland.

#### Description of holotype

Body stout; head slightly wider than long (HL/HW = 0.94); snout short, nearly mucronate in dorsal view, protruding in lateral profile; nostrils small, oval, directed anterolaterally, not protruding, and located near to anterior terminus of snout; frontal macrogland absent; loreal region slightly concave, vertical; eye large, not protruding, pupil horizontally elliptical; eye diameter equal to half the interorbital distance (ED/IOD = 0.5); cephalic crests absent; parotoid gland absent; tympanic membrane and supratympanic fold absent; without evident co-ossification of the dorsal skull elements; premaxillary, maxillary, and vomerine teeth absent; tongue narrow and elongate, posterior margin entire, posteriorly free for about two thirds of length; widely separated, small and rounded choanae; vocal sac medial subgular, with an evident external fold; without externally evident ventral humeral crest; arms and forearms robust; finger blunt, short and slightly webbed at the base; finger tips rounded; keratinized light brown nuptial pads on finger II and along the proximal medial margin of finger III; relative lengths of fingers IV>V>III = II; inner metacarpal tubercle large and rounded; outer metacarpal tubercle rounded, three times smaller than inner; subarticular tubercles rounded; several rounded supernumerary palmar tubercles spaced from one another; legs short; robust, toes shorts, unreduced; toe tips rounded; relative lengths of toes IV>III = V>II>I; inner metatarsal tubercle oval, outer metatarsal tubercle rounded, one and a half time larger than inner; subarticular tubercles rounded, divided on finger IV; supernumerary plantar tubercles small, rounded, and spaced from one another; skin of dorsum entirely covered with several small gland, tipped with keratinized spines (usually one per warts, but a few warts with more than one); ventral skin covered with small low warts; warts tipped with several small spines. Measurements of holotype (in mm) are: SVL = 18.9; HL = 6.2; HW = 6.3; ED = 2.1; IOD = 4.1; IND = 1.3; END = 2.0; THL = 7.2; TBL = 6.9; FL = 10.7.

#### Color in life

Dorsum and sides of the face dark brown (A80: M99/C90) with reddish brown spots (A70: M90/C70) in the middle of the dorsum; these reddish brown spots becoming diluted and little visible from the middle of the dorsum up to the supracloacal region (Fig 9). Body sides, between arms and legs, very dark brown (N99: A50/M20) with some white dots on glandular warts. Body sides from the armpit to the side of the face with a brown stripe (A70: M60/C50). Arms, in dorsal view, dark brown (A80: M99/C90) with an orange band (A70: M40/C10) extending from the armpit to the elbow and with small white dots on glandular warts in both brown and orange areas. Forearms, legs, hands, and feet, in dorsal view, blackish (A70: M40/C10), with small white dots on glandular warts. Fingers and toes, also in dorsal view, blackish (A70: M40/C10) tipped red (A80: M99/C50) with several white dots, on blackish area, in outer fingers and toes, and red (A80: M99/C50) with white and blackish (A70: M40/C10) in proximal areas, in inner fingers and toes. Belly black with a large spot bright red (A80: M99/C10) occupying the distal third and entirely covered with small white dots, less intense in the gular region (Fig 9). Near the cloaca, dark red (A80: M99/C40). Arms, in ventral view, bright red (A80: M99/C10) with white dots on glandular warts and with yellow (A70: M00/C00) in the proximal margin of the red. Forearms, in ventral view black with a red spot proximal—in continuum with the red of the arms, and some white dots. Legs, in ventral view, black with white dots on glandular warts and with dark red (A80: M99/C40) thighs. Hands, in ventral view, red (A80: M99/C50). Feet, in ventral view, red (A80: M99/C50) with a black diagonal line starting between the metatarsal tubercles and ending near the toe V (the outer metatarsal tubercle is a red spot on this black line). Iris black with several golden spots and with a narrow golden ring bordering the pupil (Fig 9).

#### Variation in type series

The range of measurements of adult paratypes is shown in Table 1. In dorsal view, snout shape varies from mucronate (DZUP 200, 202–3, 205–7, 227) to rounded (DZUP 199, 201, 204, 225–6). Nostrils are protruding in two specimens (DZUP 202–3). The relative length of fingers is variable: IV˃ III˃V˃II (DZUP 204); IV˃V˃III˃II (DZUP 201–2, 226–7); or IV˃V = III˃II (DZUP 199, 206–7). The relative length of toes is also variable: IV˃III˃V˃II˃I (DZUP 199, 202, 204, 207, 226–7) and IV˃V = III˃II˃I (DZUP 201). Mature males have nuptial pads on dorsal surface of finger II and proximal half of finger III. The nuptial pad spines are either strongly pigmented (DZUP 199–207, 225–7) or poorly pigmented (DZUP 201, 205), and, in some specimens, the nuptial pad spines are larger on finger III (DZUP 199–200, 202–4, 207).

Some variation is found in dorsal and ventral color patterns (Fig 10). The dorsal pattern can be of a lighter tone (DZUP 199, 202, 226), of a general brown color (A70: M99/C99) with several spots of a lighter brown color (A70: M90/C90), or can be darker (DZUP 204, 206–7, 225), of a very dark brown color (N99: A40/M50) with only a few lighter brown spots (A80: M90/C80). The subtle brownish supracloacal region is a little more conspicuous in DZUP 227 and absent in DZUP 202 and 204; in DZUP 201, however, there is a white spot in a glandular wart. The light-colored band on the arms can be broader in dorsal view (DZUP 227), somewhat smaller (DZUP 202–3), restricted to around de armpit (DZUP 225), almost imperceptible due to a darker tone (N10: A50/M20: DZUP 200, 226; or A80: M90/C80: DZUP 199, 206) or, it can be absent altogether (DZUP 201, 207). There is little variation in forearms, hands, legs, and in feet, in dorsal view, except for some specimens that have a little less white spots, the inner fingers of DZUP have a broad white proximal margin, and forearms and legs of DZUP 204 are brown (A70: M99/C99). In the ventral surface, paratypes are remarkably homogenous, with evident variation in the number of white dots (with more or fewer dots), span of the red spot and its form in the proximal edge, and in the amount of yellow. Yellow can be absent (DZUP 202), present only in the right arm (DZUP 207), and present also in the border of the ventral red spot, as a few small dots (DZUP 226–7) or as yellow dots and yellow borders (DZUP 199). Iris is variable: as the holotype but also with greenish spots (DZUP 200); also with golden and greenish spots but forming only a half ring around the pupil, with both of this colors, in the cranial border (DZUP 206, 225–6); and an entirely golden iris with black spots (DZUP 207). The iris of some specimens is very dark brown, as opposed to the black iris of most specimens.

#### Variation including referred specimens

There is sexual dimorphism in SVL (Table 1), with females being larger than males (20.3–23.7 mm, mean = 22.2 mm, N = 3 vs. 17.6–21.1 mm, mean = 19.2 mm, N = 31, for females and males, respectively).

#### Etymology

The specific epithet honors Miguel S. Milano, an environmentalist who dedicated his life to protect Brazilian nature, such as serving as the director of the “Fundação Grupo O Boticário de Proteção à Natureza” for many years.

#### Natural history

**Fig 11:**
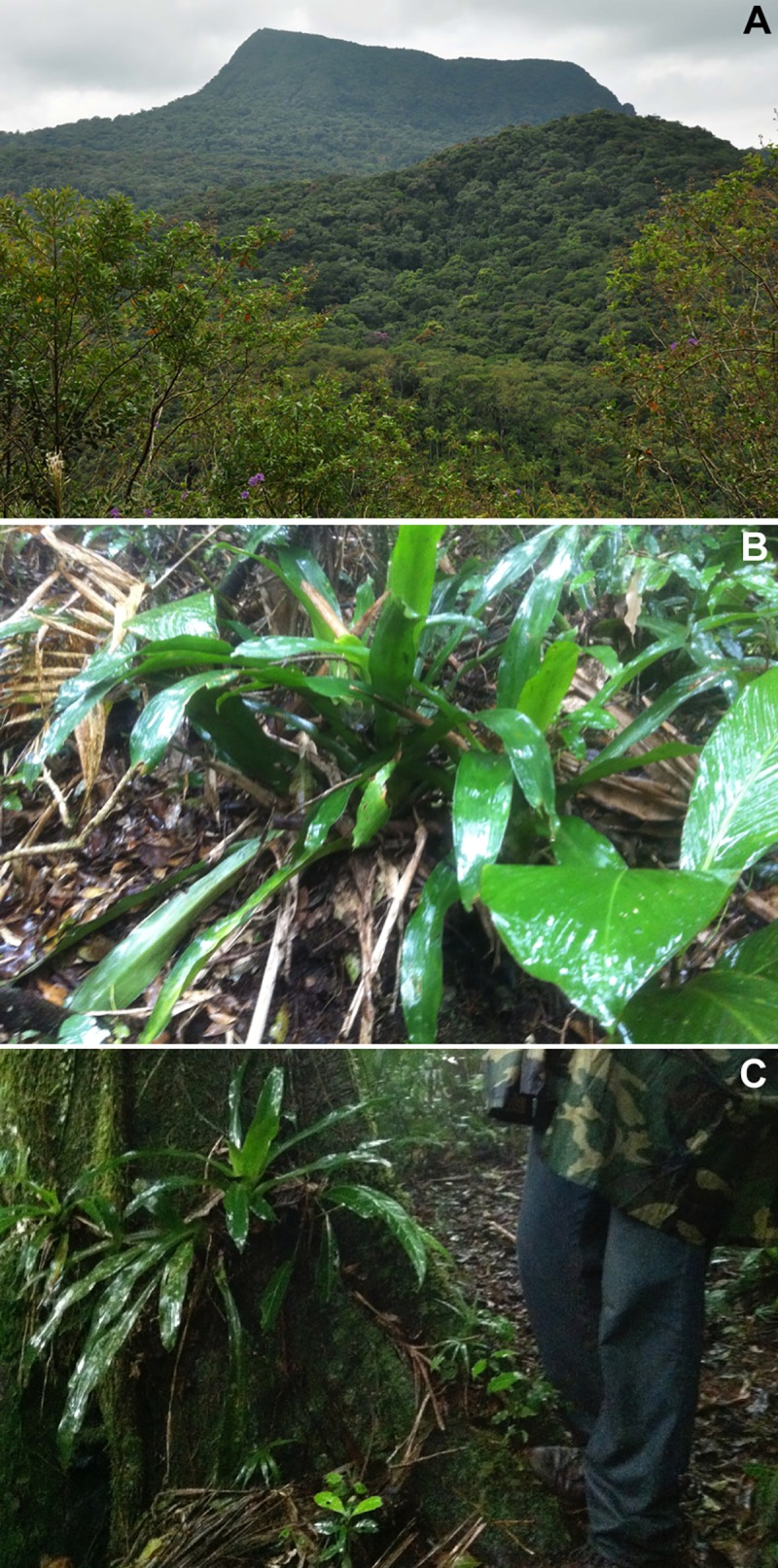
Habitat of *Melanophryniscus milanoi* sp. nov. A = Type-locality (Morro do Baú, municipality of Ilhota), around the foothills of the mountain in the top of the photograph. B = Terrestrial bromeliad where a male was calling at Morro Boa Vista (on the border between the municipalities of Jaraguá do Sul and Massaranduba). C = Epiphytic bromeliad where a male was calling in Morro Azul (on the border between the municipalities of Pomerode and Rio dos Cedros).

**Fig 12:**
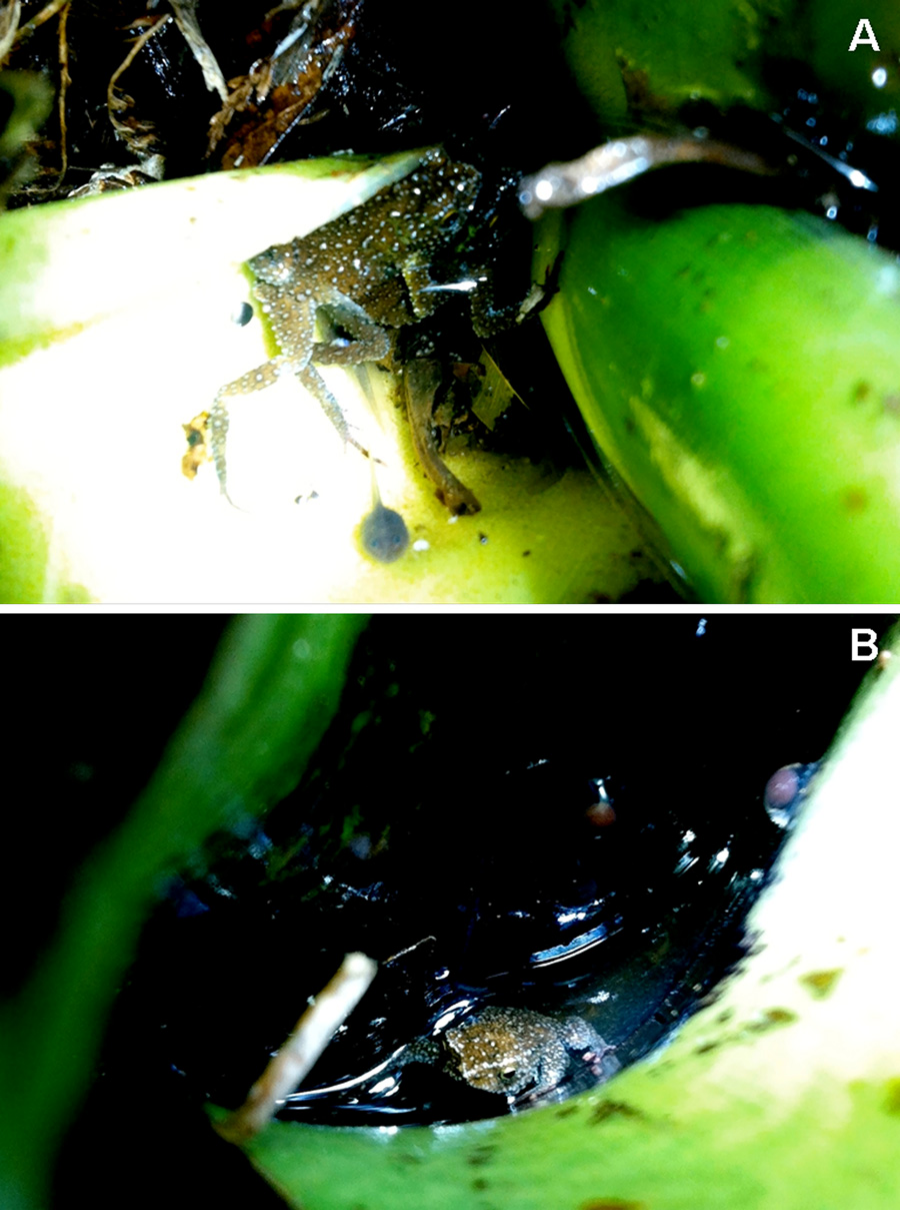
*Melanophryniscus milanoi* sp. nov. in their habitat. A = pair during amplexus—notice the tadpole in the water below. B = Adult male, in most common position of calling males—notice the two eggs, one attached to the abaxial surface of the leave and the other to the adaxial surface (right egg). (These eggs are out of the water because the water flowed out after the leaf of the bromeliad has been away for the camera approach). Both photos taken in the Morro do Cachorro (on the border between the municipalities of Blumenau, Gaspar, and Luiz Alves).

*Melanophryniscus milanoi* sp. nov. is a montane species (655–850 m a.s.l.; Fig 11A) that inhabits forests (“Floresta Ombrófila Densa Montana”) and secondary vegetation in advanced stages of regeneration. We detected males calling (in daylight), two pairs in axillary amplexus (Fig 12A), eggs, and tadpoles in terrestrial bromeliads (Fig 11B) and, rarely, in epiphytic bromeliads (in this case over fallen trunks and in trees up to 1 m high, Fig 11C). In the type locality, the species breeds in *Vriesea platynema*, *V*. *incurvata*, *Nidularium amazonicum* (= *Wittrockia smithii*), and in *N*. *procerum*, at least. We included *Vriesea altodaserrae* as an additional potential breeding site for the species, as recorded in Morro do Cachorro. In the type locality, we heard only two individuals on 23 October and several individuals three days later in terrestrial bromeliads. On 25 and 26 October we heard several individuals in terrestrial and epiphytic bromeliads in Morro Boa Vista and Morro Azul, and on 29 October we found eggs, tadpoles, calling males, and pairs in amplexus at Morro do Cachorro (Fig 12), also in terrestrial and epiphytic bromeliads. Reproductive activities were observed in the water accumulated at the base of green leaves of bromeliads, either horizontal or slightly inclined upwards (Fig 12). A tadpole was observed in the water accumulated in the young leaves of the apex of a bromeliad. Eggs were isolated from each other and were found freely in the water or attached to the adaxial and abaxial surfaces of leaves (Fig 12B).

#### Geographical distribution

The species is known from the type locality and from three additional localities of referred specimens, all of them situated between the right margin of Itapocu river and left margin of Itajaí-Açu river, northeastern Santa Catarina, southern Brazil (Fig 7). The distances between these localities are 32 km in the north-south direction and 27 km in the east-west direction.

### *Melanophryniscus xanthostomus* sp. nov.

(Figs 13–15)

**Fig 13:**
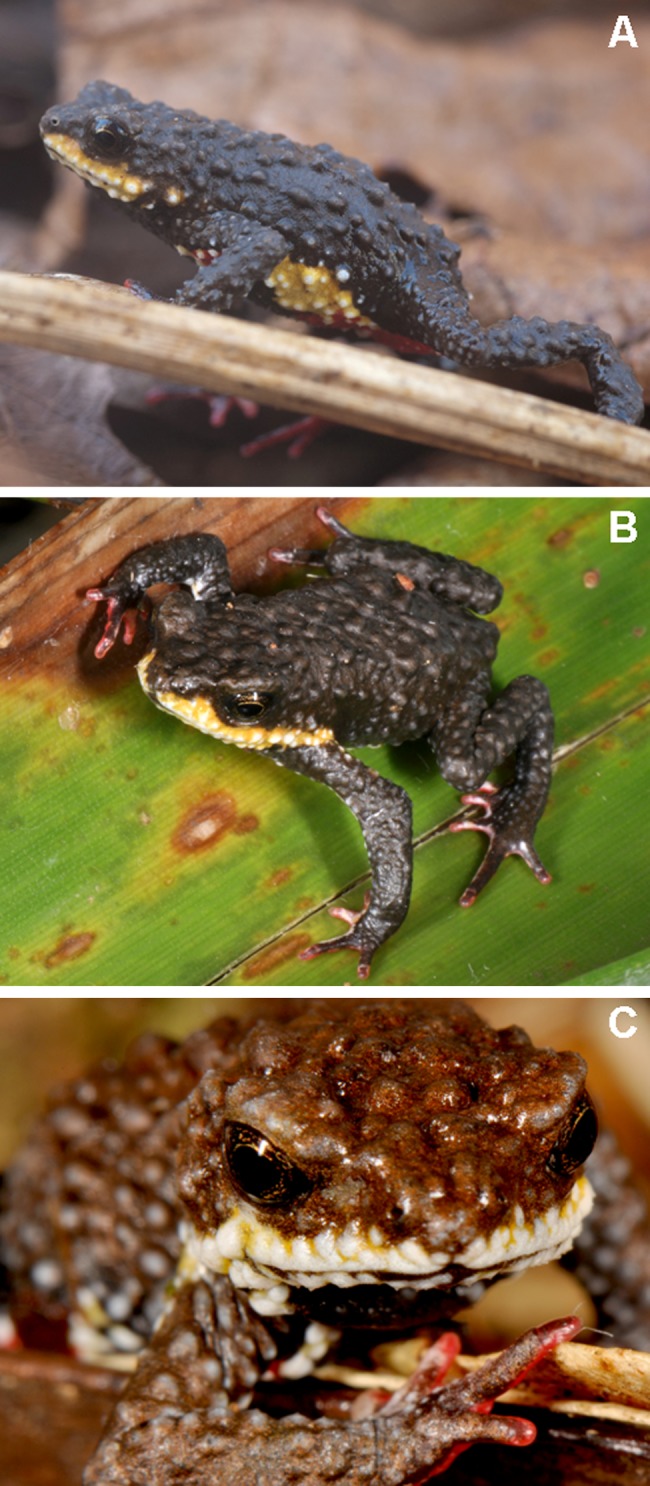
*Melanophryniscus xanthostomus* sp. nov. A = Holotype, adult male from Serra do Quiriri (DZUP 192); B = Paratype, adult male from Morro do Boi (DZUP 461); C = Paratype, adult male from Serra Queimada (DZUP 368).

**Fig 14:**
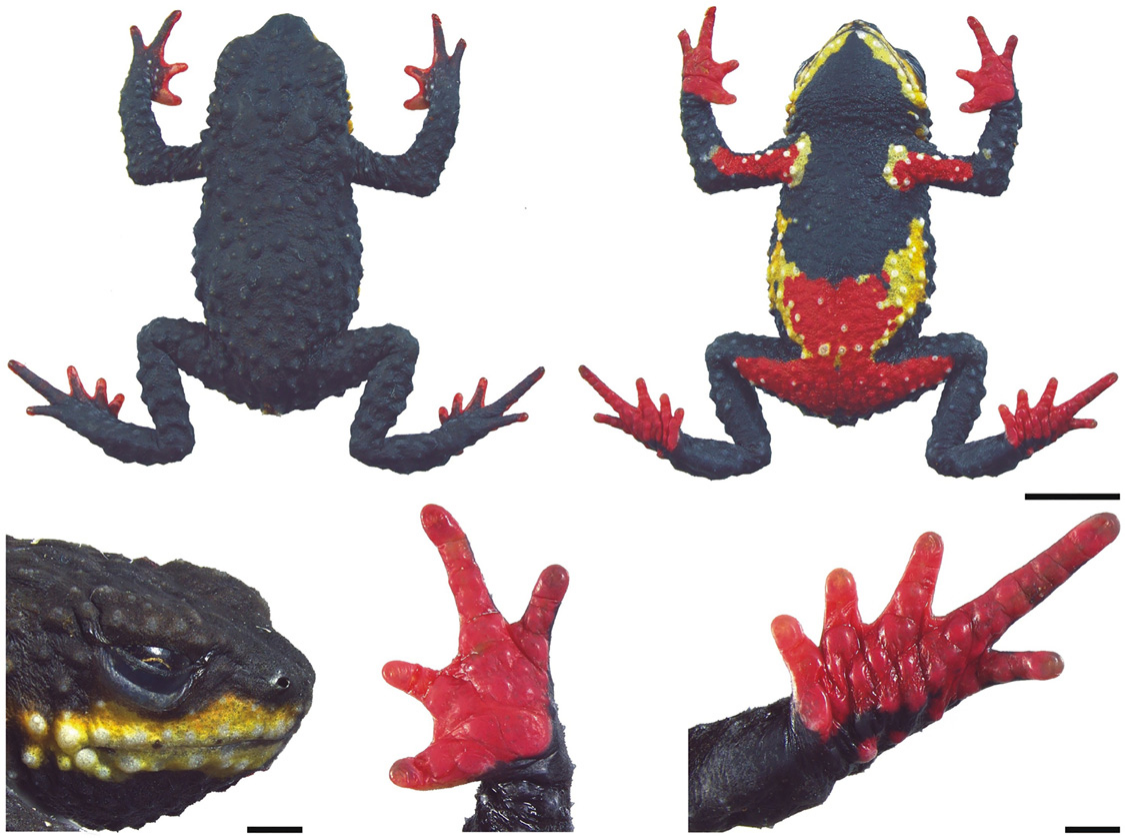
Holotype of *Melanophryniscus xanthostomus* sp. nov. (DZUP 192), adult males, a few minutes after being fixed. The lower surface of the right hand and right foot are shown on the bottom right. The scale bars on the top two photographs correspond to 0.5 cm, whereas on the bottom they correspond to 1 mm.

**Fig 15:**
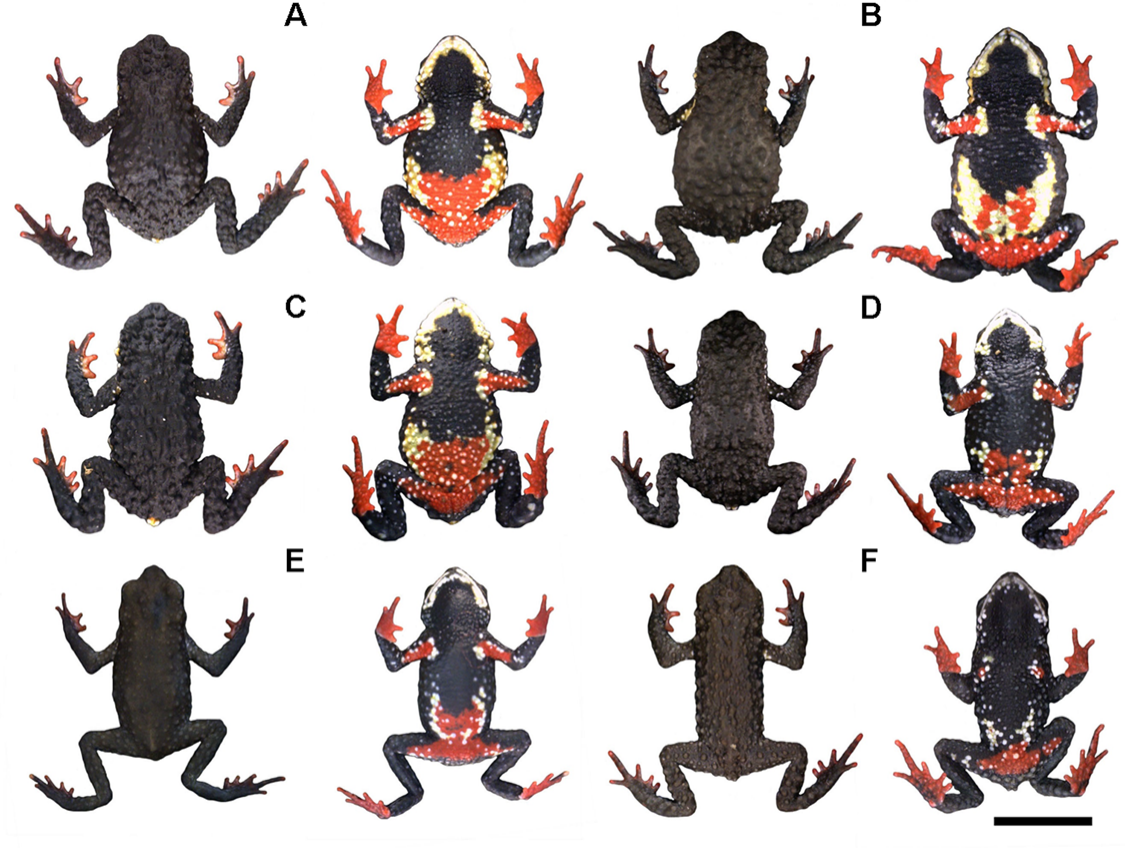
Representative variation in coloration in the type-series of *Melanophryniscus xanthostomus* sp. nov., alive, in dorsal and ventral view. A = DZUP 196 (adult female); B = DZUP 194 (adult female); C = DZUP 195 (adult male); D = DZUP 193 (adult male); E = MHNCI 9807 (adult male); F = MHNCI 9808 (adult male). The bar corresponds to 1 cm.

D. Baldo, M.R. Bornschein, M.R. Pie, L.F. Ribeiro, C.R. Firkowski & S.A.A. Morato

Urn:lsid:zoobank.org:act: 993E45FF-867B-41EC-89AB-1BA8CC9430B8

*M*. sp. 4 [24]

#### Holotype

DZUP 192 (Figs 13A and 14), male collected Serra do Quiriri (26°01’17”S, 48°59’47”W; 1,270 m a.s.l.), municipality of Campo Alegre, state of Santa Catarina, southern Brazil, on 3 February 2011 by MRB, MRP, and Dulce Carvalho.

#### Paratopotypes

DZUP 193 and 195 (Fig 15), two males, and DZUP 194 and 196 (Fig 15), two females, all with same data as holotype. All specimens collected between 1,255 and 1,275 m a.s.l. Tissue samples collected from all specimens (DZUP).

#### Paratypes

MHNCI 9806–8 (ex DZUP 189–91; Fig 15), three males collected at Condomínio Vale dos Lagos (26°08’48”S, 49°10’43”W; 930–935 m a.s.l.), municipality of Joinville, state of Santa Catarina, southern Brazil, on 23 January 2011 by MRB and SAAM. DZUP 368–70, three males collected at Reserva Particular do Patrimônio Natural Caetezal (26°06’32”S, 49°03’26”W; 1,240–1,270 m a.s.l.), Serra Queimada, municipality of Joinville, state of Santa Catarina, southern Brazil, on 26 September 2012 by MRP, LFR, and Felipe A. Cini da Silva. DZUP 461, male, and DZUP 462, female, collected at Morro do Boi (26°24”53”S, 49°13’08”W; 565 m a.s.l.) municipality of Corupá, state of Santa Catarina, southern Brazil, on 3 November 2012 by MRP, LFR, and Felipe A. Cini da Silva.

#### Diagnosis

Due to its intermediate size (SVL = 18.1–21.5 mm), *M*. *xanthostomus* sp. nov. differs from: the smaller (*M*. *vilavelhensis*, SVL = 12.8–17.2 mm, [16]; *M*. *biancae* sp. nov. SVL = 12.9–13.8 mm [see above], and *M*. *setiba*, SVL = 13.8–16.1 mm, [17]), and from the larger species of the genus (*M*. *estebani*, SVL = 25.0–34.0 mm, [19]; *M*. *admirabilis*, 29.5–40.3 mm, [14]; and *M*. *rubriventris*, SVL = 32.0–42.7 mm). As to morphology, *M*. *xanthostomus* sp. nov. is most similar to *M*. *milanoi* sp. nov. These two species can be distinguished as follows (characters of *M*. *milanoi* sp. nov. in parentheses): dorsum dark brown to black with a large yellow and white strip along the maxilla, also covering the lower half of loreal region (dorsum almost homogeneously dark brown to black; Fig 10); mandibular arch entirely covered with a large yellow and white strip (gular region spotless; Fig 10); and dorsally with medium-sized, rounded glandular warts tipped with very few spines and venter covered with medium glandular warts tipped with a spine (dorsal skin rugose, entirely covered with small to medium glandular warts and ventrally with small glands warts, both tipped with several apical spines). *Melanophryniscus xanthostomus* sp. nov. differs from the other medium-sized species that breed in Bromeliaceae, *M*. *alipioi* (SVL = 19.4–27.8 mm; characters of this species in parentheses), by its is dorsal color pattern black to dark brown with a large yellow and white strip along the maxilla, covering too the lower half of loreal region (dorsal, maxillar, and loreal areas homogeneously brown to blackish; Fig 5); ventral surface with yellow spots on chest and abdomen, and another yellow strip covering the mandibular arch (without yellow on the ventral surface; Fig 5); and rugose dorsal skin, scattered with medium-sized, glandular warts tipped with very few apical spines (dorsally rugose, scattered with large glandular warts without keratinized spines; Fig 5).

*Melanophryniscus xanthostomus* sp. nov. differs from the intermediate-sized *M*. *moreirae* (SVL = 20.6–29.1 mm) as follows (states of *M*. *moreirae* between parentheses): snout shape mucronate in dorsal view (nearly rounded); rugose dorsal skin with medium-sized glandular warts, uniformly scattered, and tipped with apical spines (smooth dorsal skin, covered with large glandular warts, devoid of apical spines in males and with only a few keratinized spines in females); dorsum dark brown to black with a large yellow and white strip along the maxilla, also covering the lower half of loreal region (dorsum dark grey to black or brownish with scattered light areas with a supracloacal white spot); arm upper surface with few small white spots (a white stripe covering the whole arm upper surface); and ventral surface with yellow spots on chest and abdomen, and another yellow strip covering the mandibular arch with a large red abdominal-femoral spot scattered with white dots (ventral pattern dark brown with bright red in the pectoral, abdominal, and femoral areas, without yellow; red spots densely dotted with small white dots in throat, gular and pectoral areas).

*Melanophryniscus xanthostomus* sp. nov. differs from small sized *M*. *setiba* (SVL = 13.8–16.1 mm) as follows (states of *M*. *setiba* between parentheses): dorsal skull elements not co-ossificated (dorsal skull elements strongly co-ossificated); fingers and toes are unreduced (reduced phalangeal formula of both hands and feet); males without humeral spine externally visible (with an evident bifurcated humeral spine); the males have a nuptial pad with several small brown-colored keratinized spines on fingers II and III (males with nuptial pad, with few enlarged, brown-colored keratinized spines at medial margin of finger II); head and dorsum skin with glandular warts tipped with apical spines (slightly granular without keratinous spines; throat and chest smooth); dorsum black to dark brown, with a large yellow and white strip in the maxilla (dorsum reddish brown to dark brown with two dorsal marks, with the first mark on the anterior region shaped like an “X”, whereas the second mark is on the posterior region and shaped like a “Ʌ”); ventral coloration black to dark brown with yellow spots on chest and abdomen and another covering the mandibular arch and with a large red abdominal-femoral spot (light orange with large dark brown blotch at midbody).

*Melanophryniscus xanthostomus* sp. nov. has a rugose dorsal skin with medium-sized glandular warts, uniformly scattered, and tipped with apical spines allowing differentiate it also from the following species: *M*. *admirabilis* (skin with large yellowish glandular warts, without apical spines); *M*. *biancae* sp. nov. and *M*. *vilavelhensis* (dorsal skin with very low glandular warts covered with few small spines); *M*. *langonei* (dorsum rugose with small to medium glandular warts, tipped with several apical spines and with several longitudinal glandular ridges); *M*. *sanmartini* (dorsum rugose, with small to medium rounded glandular warts scattered, more abundantly in the head, and tipped with several apical spines); and from several species of the *M*. *stelzneri* group (skin almost smooth, with few developed glandular warts, tipped with few and small apical spines in *M*. *estebani*, *M*. *fulvoguttatus*, *M*. *klappenbachi*, and *M*. *stelzneri*; or skin smooth with large glandular warts devoid of apical spines in *M*. *rubriventris*).

The dorsum dark brown to black with a large yellow and white stripe along the maxilla, also covering the lower half of loreal region, and few small white spots on upper arms differentiates *M*. *xanthostomus* sp. nov. from the following species: *M*. *admirabilis* (almost homogeneously green to yellowish); *M*. *alipioi* and *M*. *krauczuki* (almost homogeneously brown to black); *M*. *biancae* sp. nov. (dorsum with shades of brown color, patchily distributed or sometimes divided in side-by-side lines; Fig 3); *M*. *langonei* (light brown with the glandular ridges darker and with a white stripe on upper arms); *M*. *moreirae* (dorsum dark grey to black or brownish with scattered light areas with a white stripe on upper arms and a supracloacal white spot); *M*. *sanmartini* (dark brown with light brown spots and with a white stripe on upper arms); and from many species of *M*. *stelzneri* group (black, dark brown or dark green, with head and body covered with copper, yellow, orange or red spots in *M*. *cupreuscapularis*, *M*. *dorsalis*, *M*. *estebani*, *M*. *fulvoguttatus*, *M*. *klappenbachi*, *M*. *montevidensis*, *M*. *paraguayensis*, *M*. *rubriventris*, and *M*. *stelzneri*).

The ventral coloration dark brown to black with yellow spots on chest, abdomen and a yellow with white stripe covering the mandibular arch distinguishes *M*. *xanthostomus* sp. nov. from *M*. *admirabilis* (gular region black with large yellow glands; chest and belly black, with irregular spots pale green or yellowish); *M*. *biancae* sp. nov. (entirely yellow with scattered white dots; Fig 3); *M*. *krauczuki* (ventral of head dark brown with numerous small white dots along the mandibular arch; pectoral zone dark brown with small white dots, belly dark brown with large, irregular, and bright orange spots); and from *M*. *dorsalis* (black with red spots on chest, abdomen and with a large femoral patch).

Finally, *M*. *xanthostomus* sp. nov. is easily distinguishable from all species of the *M*. *tumifrons* group (*M*. *devincenzii*, *M*. *macrogranulosus*, *M*. *pachyrhynus*, *M*. *peritus*, *M*. *simplex*, *M*. *spectabilis*, and *M*. *tumifrons*) due to the lack of frontal macrogland.

#### Description of holotype

Body stout, head wider than long (HL/HW = 0.87), less than one third of SVL (HL/SVL = 0.29); snout short, slightly acuminated in dorsal and lateral views; nostrils small, oval, directed anterolaterally, not protruding, and located almost at tip of snout; frontal macrogland absent; loreal region slightly concave, vertical; eye large, not protruding; eye diameter slightly less than half of the interorbital distance (ED/IOD = 0.44); pupil horizontally elliptical; cephalic crests absent; parotoid gland absent; tympanic membrane and supratympanic fold absent; without evident co-ossification of the dorsal skull elements; premaxillary, maxillary, and vomerine teeth absent; tongue narrow and elongate, posterior margin entire, posteriorly free for about two thirds of length; widely separated, small and rounded choanae; vocal sac medial subgular, without evident external fold; without externally evident ventral humeral crest; arms and forearms robust; finger blunt, short and slightly webbed at the base; finger tips rounded; unpigmented nuptial pads along the proximal medial margin of finger II, and very few on finger III; fingers relative lengths IV˃V˃III = II; inner metacarpal tubercle small, rounded, poorly distinguished, outer metacarpal tubercle flat and rounded, four times greater than the inner; subarticular tubercles large and rounded, divided on finger IV; few large and rounded supernumerary palmar tubercles; legs short, robust; tarsus particular bulky; toes shorts, unreduced; slightly webbed at the base; toe tips rounded; relative lengths of toes IV˃V˃III˃II˃I; inner metatarsal tubercle oval; outer metatarsal tubercle rounded, three times larger than inner; subarticular tubercles medium-sized and rounded, divided on finger IV; supernumerary plantar tubercles large, rounded, and spaced from one another; dorsum skin covered with medium-sized rounded glandular warts, tipped with several spines (from 1 to 11); post commissural zone with bigger and numerous conical glandular warts; ventrally covered with low glandular warts tipped with a spine (exceptionally two). Measurements of holotype (in mm) are: SVL = 21.3; HL = 6.2; HW = 7.1; ED = 2.0; IOD = 4.6; IND = 1.8; END = 1.7; THL = 8.4; TBL = 7.3; FL = 11.9.

#### Color in life

Dorsum and body sides very dark brown (N99: A30/M50; Fig 14). Body sides, between arms and legs, with few small and medium white dots on glandular warts and with a large yellow (A70: M20/C10) spot that extends into the belly. Body sides from the armpit to the mouth with some white and darker yellow (A70: M50/C10) dots. Along the maxilla with a large darker yellow (A70: M50/C10) and white strip, also covering the lower half of loreal region. Mandibular arch with a yellow (A70: M20/C10) and white strip. Belly black with a large bright red spot (A80: M99/C10) occupying the distal quarter and with one large yellow (A70: M20/C10) spot, in each side of the belly, that reaches the sides of the body (Fig 14). There are some small and medium white dots on glandular warts in the red and yellow regions, two small white dots in the black region, and on small yellow spot in the red region. Near the cloaca, dark red (A80: M99/C40). Arms and legs, in dorsal view, very dark brown (N99: A30/M50); there are few small white dots on glandular warts in the arms. Hands and feet, in dorsal view, very dark brown (N99: A30/M50), with small white dots in hands. Fingers and toes, also in dorsal view, very dark brown (N99: A30/M50) tipped red (A70: M90/C30) and with white narrow margins in the proximal borders of inner digits. Arms, in ventral view, bright red (A80: M99/C10) with white dots on glandular warts and with yellow (A70: M20/C10) in the proximal margin of the red, already in the belly. Forearms, in ventral view, black with a red spot proximal in right forearm—reaching the red of the arm, and some white dots. Legs, in ventral view, black with dark red (A80: M99/C40) thighs. Hands, in ventral view, red (A70: M90/C30). Feet, in ventral view, red (A70: M90/C30) with a black diagonal line starting between the metatarsal tubercles and ending near the toe V (there is two red spots in tubercles in this black line). Supracloacal region orange (A70: M40/C00) with three white dots on the border of the orange spot. Iris black with several golden spots and with a narrow golden ring bordering the pupil (this ring is complete in the superior half and incomplete in the inferior half of the eye; Fig 14).

#### Variation in type series

The range of measurements of adult paratypes is shown in Table 1. There is sexual dimorphism in the SVL (Table 1), with females being slightly larger than males (20.5–21.5 mm, mean = 21.1 mm, N = 3 vs. 18.1–21.5 mm, mean = 20.4, N = 9). Nostrils slightly protruding in some specimens (MHNCI 9807, DZUP 193, 195–6). The relative length of fingers varies from IV˃V˃III˃II (MHNCI 9806–8, DZUP 192, 194–5) to IV˃V˃III = II (DZUP 196). The relative length of toes is also variable: IV˃III = V˃II = I (MHNCI 9808); IV˃III˃V˃II˃I (MHNCI 9806); IV˃V = III˃II˃I (MHNCI 9807); IV ˃V˃ III ˃II˃I (DZUP 194–6); and IV ˃V˃ III ˃II = I (DZUP 192). Mature males have nuptial pads on dorsal surface of finger II and proximal half of finger III (only on finger II in DZUP 370). The nuptial pad spines range from strongly pigmented (MHNCI 9806, 9808, DZUP 193, 368–9) to unpigmented (DZUP 192, 195–6, 370). There are also variations in the quantity and size of glandular warts, with the specimen with smoother skin being MHNCI 9807 (Fig 15E).

The coloration patterns are variable (Fig 15). Between paratopotypes there is little variation: Dorsum and arms and legs, in dorsal view, can be black (DZUP 195; Fig 15C); supracloacal colored spot can be more conspicuous (DZUP 195; Fig 15C) or inconspicuous, differing little from the surrounding color (DZUP 194; Fig 15B); arms, in dorsal view, may have a small darker yellow (A60: M40/C00) spot (DZUP 195) or a small bright yellow (A60: M10/C00) spot (DZUP 194; Fig 15B); body sides, between armpit and the mouth, can be more intensely colored with yellow (DZUP 194–5) or completely colored with yellow and white, making a continuous strip from mouth until armpit (DZUP 196); the colored strip on maxilla and mandibular arch can be broader (DZUP 195–6; Fig 15C), more whitish (DZUP 193; Fig 15D), or interrupted on the chin (*e*.*g*. DZUP 194; Fig 15B); and belly can show less yellow (DZUP 193; Fig 15D), less red (DZUP 194; Fig 15B), and more white dots (DZUP 195–6; Fig 15C and 15A). Paratypes from the Reserva Particular do Patrimônio Natural Caetezal are similar of the specimens from the type-locality with less amount of red and yellow on belly, and with narrow colored strip on mouth (DZUP 193), with some species being less colored with yellow on belly and with an even narrower colored strip in mandibular arch (DZUP 368; Fig 13C), but having more white dots of any of those specimens. The dorsum of specimens from Reserva Particular do Patrimônio Natural Caetezal resembles the brownish paratopotypes DZUP 194, not only by the brown dorsum but also by the less conspicuous supracloacal colored spot. In the case of the paratypes of Morro do Boi, the specimen DZUP 461 (Fig 13B) is noteworthy for being one of the few individuals with its colored stripe being nearly continuous from the mouth to the armpit. Paratypes from Condomínio Vale dos Lagos are the most variable among the entire type-series: dorsum and upper surface of arms and legs are of a distinct tone of very dark brown (N99: A99/M80) in MHNCI 9807 (Fig 15E); supracloacal colored spot is missing; white dots on the dorsal region of the arms can be missing (MHNCI 9806–7; *e*.*g*. Fig 15E) or be accompanied of a small white spot in proximal extreme (MHNCI 9808; Fig 15F); white dots on the dorsal region of hands can be missing (MHNCI 9806–7; *e*.*g*. Fig 15E); colored strip on maxilla and mandibular arch narrow and less conspicuous, being discontinuous in all its extension in MHNCI 9808 (Fig 15F); belly can show numerous white dots and only a small amount of yellow; and arms and legs can be practically devoid of red. Iris is variable: as the holotype but forming a complete ring around the pupil (DZUP 461; Fig 13B); with golden and orange spots and forming a golden half ring around the superior margin of the pupil (DZUP 368; Fig 13C); with golden and greenish spots and forming a complete ring around the pupil with both of this colors (MHNI 9806); and with golden and greenish spots and forming a half ring around the superior margin of the pupil with both of this colors (MHNI 9807–8). The iris of some specimens is very dark brown, as opposed to the black iris of most specimens.

#### Etymology

The specific epithet stems from the Greek words *xanthos* (yellow) and *stoma* (mouth), indicating one of the most obvious diagnostic characters of the new species.

#### Natural history

**Fig 16:**
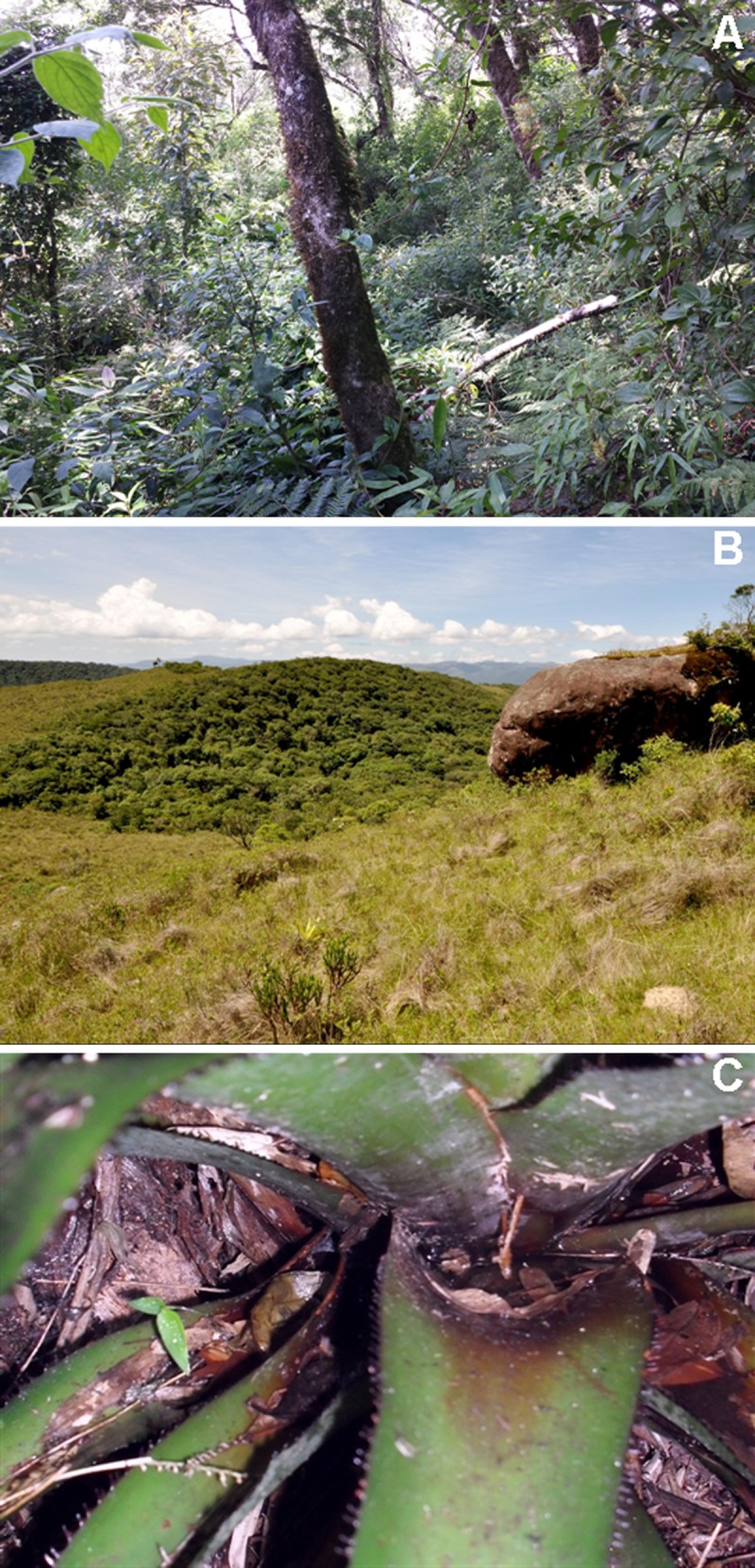
Habitat of *Melanophryniscus xanthostomus* sp. nov. A = Type-locality (Serra do Quiriri, municipality of Campo Alegre)—this forest had a fire a few years before that killed many trees, leading to intense sunlight inside and the occupation of dense vegetation in the understory. B = Cloud forest, habitat of the species at Reserva Particular do Patrimônio Natural Caetezal, top of the Serra Queimada (municipality of Joinville). C = Terrestrial bromeliad (*Aechmea distichantha*) where a male was calling in the water tank in the center of the photo (municipality of Campo Alegre).

*Melanophryniscus xanthostomus* sp. nov. is a montane species (565–1275 m a.s.l.) that inhabits cloud forest (“Floresta Ombrófila Densa Altomontana”; Fig 16A and 16B), montane forest (“Floresta Ombrófila Densa Montana”), and the transition of montane forest with the Araucaria Forest (“Floresta Ombrófila Mista Montana”). We detected males calling (in daylight) and eggs in terrestrial bromeliads (*Aechmea distichantha*, *A*. *gamosepala*, *Vriesea incurvata*, *V*. *philippocoburgi*, among other unidentified species; Fig 16C). In the type locality, we also detected males calling on a dead bamboo (*Merostachys multiramea*, Poaceae), some of which partially broken and without water inside, and a pair in amplexus walking on the ground. We heard males calling on 26 September, 3 November, 23 January, and on 3 February, and we found eggs on 27 January and on 3 February. The reproductive activities were observed in the water accumulated at the base of green leaves of bromeliads, either horizontal or slightly inclined upwards. Eggs were isolated from each other and were found freely in the water or attached to the adaxial and abaxial surfaces of leaves. In one bromeliad, we found 10 eggs (one of which dead and undeveloped), with three, three, and four eggs per leaf axis. Two of these eggs were out of the water. In another bromeliad, we found 11 eggs, 10 of which in the water retained in the axis of only a single leaf; three eggs were dead and undeveloped.

#### Geographical distribution

The species is known from the type locality, three additional localities of paratypes, and an additional locality where eggs (identity confirmed by genetic analysis) were collected, namely Laranjeiras (26°06’42”S, 49°10’36”W; 860 m a.s.l.), municipality of Campo Alegre, state of Santa Catarina, southern Brazil, on 27 January 2011 by MRB and SAAM, all in northeastern state of Santa Catarina, southern Brazil (Fig 7). The distances between these localities are approximately 44 km in the north-south direction and 21 km in the east-west direction.

## Discussion

### Phytotelm-breeding

The life-history strategy of phytotelm-breeding is associated with a variety of differences in relation to its alternatives in *Melanophryniscus*. For instance, we collected a female of *M*. *alipioi* with 16 ovarian eggs (DZUP 344), which is less than half of the observed clutch size of *M*. *moreirae* (33–70 eggs; [29, 30]) and considerably fewer than the hundreds of eggs per clutch recorded in other species (56–223 in *Melanophryniscus* sp. [31], 80–351 in *M*. *stelzneri* [32, 33], 105 in *M*. *dorsalis* [7], 122 in *M*. *montevidensis* [34], and 294–401 in *M*. *krauczuki* [11]), possibly indicating an adaptation for phytotelm-breeding. The reduced number of eggs recorded in a water tank (1–9) suggests that a complete clutch is laid in more than one tank. If so, the pair in amplexus might have to move from one tank to another, which might result in eggs being released accidentally. This could be the cause of the records of eggs out of the water and in apparently unusual locations.

The few cases of reproduction by species of *Melanophryniscus* in epiphytic bromeliads suggest that terrestrial adults have limited access to plants that are high above the ground level. The *Melanophryniscus* species that reproduce in bromeliads use a large number of plant species, from several genera, suggesting a lack of specificity for the species and even for a given genus of this family. In addition, the use of fallen dead bamboo as a breeding site seems to be an opportunistic strategy, since bamboos die after reproduction, which takes place at an interval of decades [35, 36]. Although we did not see *M*. *xanthostomus* sp. nov. calling in a water tank inside of a broken bamboo, we observed males of a population of *M*. *cf*. *alipioi* calling exactly in this condition (MRB pers. obs.).

### Conservation

It is possible that *M biancae* sp. nov. is widely distributed in marshes associated with grasslands across the entire Serra do Quiriri, where it was recorded, and also in the adjacent region of Serra do Araçatuba, in Paraná. However, these herbaceous habitats encompass a relatively small area in terms of their extent of occurrence (4.742 ha, of which 3.503 ha in the Serra do Quiriri). In addition, this region has experienced increasing anthropogenic impacts, such as the loss of habitat quality as a consequence of regular burning, cattle grazing, and intense invasion of exotic trees of *Pinus* spp. (Pinaceae). Disturbances also include replacement of natural areas due by *Pinus* spp. plantations in Paraná, and the construction of small dams and kaolin mining in Santa Catarina. Indeed, there is mining activity only 28 m away from the marsh of the type locality of the species. Burning and grazing by livestock can be particularly critical for the reproduction of the species, given that they eliminate and also damage *E*. *ligulatum*, the plant species used by *M biancae* sp. nov. as a breeding site. Consequently, *M*. *biancae* sp. nov. fits the criteria to be considered as “Endangered” (criteria B.1.a.b.(i, ii, iii)).

We estimate an extent of occurrence of *M*. *milanoi* sp. nov., in montane areas to about 77 km westward from the western locality of its occurrence, in the transition of Atlantic Rainforest and Araucaria Forest, in 307,060 ha. Considering this area of extent of occurrence, the species fits the criteria to be considered as “Endangered” (criteria B.2.a.b.(iii) and B.1.a.b.(i, ii, iii), respectively). However, due to the possibility that it has a larger extent of occurrence, which would cause it to be classified as “Vulnerable” or even as a not threatened species, we propose to classify the species as “Data Deficient”.

*Melanophryniscus xanthostomus* sp. nov. is in a similar condition as *M*. *milanoi* sp. nov.: it has a small estimated extent of occurrence, which has been exposed to many disturbances of anthropogenic origin, such as deforestation and forest retraction due to fires. In addition, it has been recorded as occurring in “no more than five locations”, which would lead the species to be categorized as “Endangered” (B.1.a.b. criteria (i, ii, iii)). Yet, but we prefer to wait for new distribution data before proposing this status of conservation and for now we also propose to classify the species as “Data Deficient”.

As management actions to protect *M biancae* sp. nov., we tentatively propose the following measures: (1) the species should be incorporated into the Brazilian list of endangered species and (2) the “Plano de Ação Nacional para a Conservação dos Répteis e Anfíbios do Sul do Brasil”; (3) the owners of *Pinus* spp. plantations in the Serra do Araçatuca, a possible area of occurrence of *M biancae* sp. nov. within the “Área de Proteção Ambiental de Guaratuba”, should be required by the “Instituto Ambiental do Paraná” (the environmental agency responsible for the region) to manage the invasion of this tree in this *Serra*; and (4) the “Fundação de Amparo à Tecnologia e Meio Ambiente” (FATMA—the environmental agency responsible for the region) should not allow the plantation of *Pinus* spp. in Serra do Quiriri, (5) FATMA should also stop issuing permits for new kaolin mining in this area, and (6) FATMA should also require the mining company to study the conservation status of the new species at the type locality before evaluating any requests for license renewal for mining operations.

We highlight that the montane habitats of the new species are also occupied by three additional frog species potentially under threat of extinction, specifically *Brachycephalus quiririensis* at Serra do Quiriri (which includes the type localities of *M biancae* sp. nov. and *M*. *xanthostomus* sp. nov.), *B*. *mariaeterezae* at Serra Queimada (another locality of occurrence of *M*. *xanthostomus* sp. nov.), and *B*. *fuscolineatus* at Morro do Baú (type locality of *M*. *milanoi* sp. nov.) [37, 38, 39]. This reinforces the need for conservation initiatives and additional research on montane regions of the Atlantic Rainforest, whose degree of endemicity can be very high, and yet their biodiversity is still poorly understood.

## Appendix I

Additional specimens examined (institutional abbreviations as in Materials and methods).

*Melanophryniscus admirabilis*.*—*BRAZIL. Rio Grande do Sul: Perau de Janeiro, municipality of Arvorezinha: MCP 7923 (holotype).

*Melanophryniscus alipioi*.*—*BRAZIL. Paraná: Capivari Grande (25°07’49”S, 48°49’15”W), Serra do Capivari, municipality of Campina Grande do Sul (topotypes): DZUP 209, 284–93, 318–9; Capivari Mirim (25°08’38”S, 48°50’35”W), Serra do Capivari, municipality of Campina Grande do Sul: DZUP 294–7, 341–4, 352–5, 361.

*Melanophryniscus atroluteus*.*—*BRAZIL. Rio Grande do Sul: Itaquí: MZUSP 814, 55577 (syntypes). ARGENTINA. Corrientes: Ituzaingó, Rincón Santa María: MLP DB 2292–3, 2394.

*Melanophryniscus cambaraensis*.—BRAZIL. Rio Grande do Sul: Cambará do Sul, Fortaleza dos Aparados: MCP 00997, 02770–1, 02784, MRCN 09797 (holotype), MNHN 04993 (paratype), MRCN 09752–3, 09755, 09757–60 (paratypes), 09762–88, 09790–2, 09794, 09799–813, 09895, 13456–62, 13466–77; São Francisco de Paula: MNRJ 04251–2, 04254; São Francisco de Paula, Floresta Nacional de São Francisco de Paula: CFBH 2954–6, MRCN 13473, MNRJ 21091–4.

*Melanophryniscus cupreuscapularis*.*—*ARGENTINA. Corrientes: Capital: Corrientes: CHUNAM 539, MFA-ZV.H 386–8; Perichón: HB 070, 072, 080,124–5, 132, 752, MFA-ZV.H 069, 286; MLP DB 1053–5, 1760–2, 3425, 3537, MFA-ZV.H 286.

*Melanophryniscus devincenzii*.—URUGUAY. Rivera: La Palma: Rubio Chico, near Subida de Pena: Cuchilla Negra: MNHN 01675 (holotype).

*Melanophryniscus dorsalis*.*—*BRAZIL. Rio Grande do Sul: Torres: MCP 00233–44, 00246–50, 00252–65, 00319–20, 06446–7, 06524–8, MNRJ 03022, 03037, 27342–4, 27346, MZUSP 021710, 102606–8.

*Melanophryniscus estebani*.*—*ARGENTINA. San Luís: Coronel Pringles: La Carolina: MACN 28877–931; between La Carolina and Inti Huasi: MACN 35406–20.

*Melanophryniscus fulvoguttatus*.*—*BRAZIL. Mato Grosso do Sul: Maracaju: MLP DB 4096–8, MNRJ 04220, 04222–30, 04232–7, 04239–44. PARAGUAY: Amambay: Estancia San Juan, 20 km NE from Bella Vista: MHNP 4487.

*Melanophryniscus klappenbachi*.*—*ARGENTINA. Chaco: 1° de Mayo: Río Tragadero: MFA-ZV.H 413–4; San Fernando: Arroyo Palometa, Ntl. Road 11: MNHN 1496 (holotype).

*Melanophryniscus krauczuki*.*—*ARGENTINA. Misiones: Candelaria: Ñu Pyahú: Prov. road 3, 3.5 km from Nat. road 12: MLP DB 0744 (holotype); FML 10124–5; MACN 37141–2; MLP DB 0719–20, 0724–7, 0730–2, 0739–40, 0742–3, 0745–6, 0750–1, 0756–8, 0766, 0768–9, 0772, 0775, 0777, 0780, 0783–6, 0807, 0827–8, 0831, 0970–6, 1900–2 (all paratypes).

*Melanophryniscus macrogranulosus*.—BRAZIL. Rio Grande do Sul: Morro da Gruta, Porto Fagundes, Porto Colônia, Dom Pedro de Alcântara: MRCN 01694 (holotype), 01693, 1695–9, 01701–2 (paratypes), 13818, 13820; Maquiné, Barra de Ouro: ZUFSM 3920.

*Melanophryniscus montevidensis*.*—*URUGUAY. Montevideo: Barra de Santa Lucía: CENAI (MACN) 03263, 03281; Montevideo: FML 00039. Rocha: Cabo Polonio CENAI (MACN) 06337, 07118, MZUSP 055618–9.

*Melanophryniscus moreirae*.*—*BRAZIL. Rio de Janeiro: Parque Nacional do Itatiaia: CENAI (MACN) 8732 (paratype), MLP 1071, 1804–5, MZUSP 13613–20.

*Melanophryniscus pachyrhynus*.—BRAZIL. Rio Grande do Sul: MZUSP 000752 (paralectotype); Piratini, ZUFSM 4141; São Lourenço do Sul, MZUSP 0756 (lectotype); Caçapava do Sul, MCP 3525; Dom Feliciano, ZUFSM 2868, 2874, 2896, 2902, 2917, 2922, 2982–4, 3753–61. URUGUAY: Maldonado: Cerro de las Ánimas, MNHN 05476 (holotype), MNHN 0392 (paratype of both *M*. *sanmartini* and *M*. *orejasmirandai*), MNHN 04383–4 (paratypes of *M*. *orejasmirandai*).

*Melanophryniscus paraguayensis*.—PARAGUAY. Central: Municipalidad de Mariano Roque Alonso, Urbanización Surubu’í: MLP DB 6763–4, 6766–8.

*Melanophryniscus rubriventris*.*—*ARGENTINA. Jujuy: Manuel Belgrano: Capillas: FML 01520, 04947; Salta: Orán: San Andrés: FML 00173 (holotype).

*Melanophryniscus sanmartini*.*—*URUGUAY. Lavalleja: Villa Serrana, near Arroyo Aiguá dam: MNHN 1676 (holotype); Salto del Penitente MNHN 9376–7; Rocha: Santa Teresa: MNHN 9378.

*Melanophryniscus simplex*.—BRAZIL. Rio Grande do Sul: Boca da Serra, São Joaquim, near to Bom Jardim do Sul: MZUSP 035599 (holotype), MNRJ 25943–5 (paratypes), MZUSP 035596, 035600–4 (paratypes).

*Melanophryniscus spectabilis*.—BRAZIL. Santa Catarina: Nova Teutonia: FML 03496–1/10, MLP 1802–3, MNRJ 25936–42 (paratypes), MZUSP 009409–20, 009440, 009447–59, 009470–73, 009475–84, 009486–92, 009494–5, 009498–501, 009508–9 (paratypes), 009424 (holotype), MZUSP 102163–99; Pequena Central Hidrelétrica (PCH), Xavantina: CFBH 18269–73; 18276–8.

*Melanophryniscus stelzneri*.*—*ARGENTINA. Córdoba: FML 00021, MZUSP 016330; Calamuchita: Athos Pampa: Río Reartes: CENAI (MACN) 04125–6; Calamuchita: Calmayo: CENAI (MACN) 03324; Calumuchita: Estancia El Sauce: MACN 07295–300, 07758, 08000, 08000:1.

*Melanophryniscus tumifrons*.—BRAZIL. Rio Grande do Sul: BMNH 1947–2–1461 (holotype).

*Melanophryniscus vilavelhensis*.*—*BRAZIL. Paraná: Parque Estadual de Vila Velha (25°14’50”S, 50°00’17”W), municipality of Ponta Grossa: MHNCI 4873 (holotype), 4874–84 (paratypes).

*Melanophryniscus* sp.—BRAZIL. Paraná: Gigante (25°27’29”S, 48°55’42”W), Serra do Marumbi, municipality of Morretes: MN 4993–4; Olimpo (25°27’13”S, 48°55’13”W), Serra do Marumbi, municipality of Morretes: MN 4253.
